# Facilitation and competition deconstructed: a mechanistic modelling approach to the stress gradient hypothesis applied to drylands

**DOI:** 10.1038/s41598-024-52447-z

**Published:** 2024-01-25

**Authors:** Rubén Díaz-Sierra, Max Rietkerk, Mart Verwijmeren, Mara Baudena

**Affiliations:** 1 Mathematical and Fluid Physics Department, Faculty of Sciences, Universidad Nacional de Educación a Distancia, UNED, 28040 Madrid, Spain; 2 Section Environmental Sciences, Copernicus Institute of Sustainable Development, Utrecht University, Utrecht, The Netherlands; 3 Centre for Complex Systems Studies, 4th Floor Minnaert Building, Leuvenlaan 4, Utrecht, The Netherlands; 4 National Institute for Public Health and the Environment (RIVM), Bilthoven, The Netherlands; 5 Institute of Atmospheric Sciences and Climate (CNR-ISAC), National Research Council of Italy, Corso Fiume 4, 10133 Torino, Italy; 6 National Biodiversity Future Center, 90133 Palermo, Italy

**Keywords:** Ecological modelling, Plant ecology, Population dynamics

## Abstract

Facilitative interactions among species are key in plant communities. While experimental tests support the Stress Gradient Hypothesis (SGH) as an association between facilitation and stress, whether the shape of net effects along stress gradients can be predicted is controversial, with no available mathematical modelling approaches. We proposed a novel test, using a modification of the R* model to study how negative and positive partial effects of plant interactions in drylands combine along two common stress gradients. We modelled different interactions: competition for water and light, amelioration of soil infiltration and/or grazing protection, obtaining that intensity and importance of facilitation did not generally increase along stress gradients, being dependent on the interaction type. While along the water stress gradient net interactions became more positive, reaching a maximum and then waning again, various outcomes were observed along the grazing gradient. Shape variety was mainly driven by the various shapes of the partial positive effects. Under resource stress, additive interaction effects can be expected, whereas when including grazing, the effects were non-additive. In the context of the SGH, deconstructing the effect of positive and negative interaction in a pairwise mechanistic models of drylands does not show a unique shape along stress gradients.

## Introduction

Plant-plant interactions are crucial in structuring plant communities and shaping their responses to environmental or biotic changes^1–5^. Classical ecological theory focused on how competition (i.e., net competitive interactions) affects the composition, diversity, stability, susceptibility to invasive species, and resilience of plant communities^6–12^. Plant-plant facilitation (i.e., net facilitative interactions) has received much attention in the last three decades, during which its role in plant communities has been emphasized^13^ with a growing body of experimental support^14–18^ and theoretical approaches^19–24^.

Switching between prevalent positive and negative effects of the interactions is an important property that can lead to changes in community and ecosystem resilience and stability^25,26^. Understanding the changes of net-interactions at different stress levels is important to help predicting the impact of changing climate and land-use on biodiversity and productivity, in preventing ecosystem degradation, and in supporting ecosystem restoration practices^27–30^.

The Stress Gradient Hypothesis (SGH)^13^ is an influential hypothesis that originated in ecology as an experimental conjecture and has evolved into a valuable tool, also used in restoration studies^23,31–33^. Additionally, the SGH has been increasingly applied to other living systems^34^, such as microbial and microbiome systems where it has garnered experimental and modeling support^35–39^, despite some divergent findings^40^. Particularly, the SGH has been fundamental in promoting discussion and inspiring research about the relationship between plant facilitation and stress. The SGH postulates that the net effect of interactions in plant communities changes from negative (competition) to positive (facilitation) with increasing stress^13,41^. At a general level the SGH is considered a ‘general rule of thumb’^42^, ‘general didactic model’^42^ or a ‘conceptual framework’ ^43,44^, and it has contributed to recognize the role of positive interactions in ecological and evolutionary dynamics. Such perspective typically relies on comparing the performance of target plants, with and without neighbors, in two situations, low vs high stress, and has shown the association of high stress levels and facilitation^45^.The validity of the SGH as a general hypothesis has been largely assessed by a significant body of experimental data, which support it^33,45–48^.

However, at a more detailed level of analysis, i.e. when characterizing the SGH “shapes”, defined as qualitative changes of the net effect of the interactions along stress gradients^19,41,49^ many studies point out to the difficulty of establishing a common qualitative behavior of net facilitation along stress gradients^42,50–53^. For this reason, variations to the SGH have been proposed, mostly to introduce that facilitation may wane under extreme stress^54^ or under a combination of stressors. The proposed “shapes” of the SGH^55^ can be either hump-shaped, increasing with saturation or with a collapse, or switching to competition at high stress levels^3,49,55–58^.

Before continuing, we need to define a few key concepts as used in the text. In a community or ecosystem, the different mechanisms of interaction (“interactions” in the following) and their dynamics lead to a *net* interaction “effect”, measured as the consequence of the interactions, although these terms are not free from controversy^59,60^ (see Supplementary S1 for a more precise definition of them in this paper). Typically, the net effects of the interactions between two plants are studied by measuring the performance of a target plant, the *protégé* or beneficiary, with and without another plant, the *nurse* or benefactor^61^. The performances with and without the nurse are compared using indices that estimate the intensity and/or the importance of the interactions (e.g. Refs.^24,47,55,62,63^).

The SGH postulates a general prediction, which may vary in detail depending on the perspective, of how the net effects vary along stress gradients, in the attempt to extract a general property of the community or ecosystem studied, emerging from the actual mechanisms of interactions. Here, non-linear dynamics are likely to play a role^43^. Notwithstanding, early works^41^ assume that the net effects of the interactions can simply be decomposed into the sum of partial positive and negative effects (with different signs). Such an additive perspective helped focusing the characterization of positive and negative effects along gradients, see e.g. Refs.^19,64,65^. However, the underlying decomposition relies on the *separability* of the partial effects, i.e. several concurring causes have independent, additive, effects, as would occur in linear models. Yet, in general, such separability is not satisfied in non-linear systems, and it cannot be assumed to occur when growth, consumption or death rates are likely non-linear functions of biotic or abiotic conditions^43^. For certain interactions, these ‘partial effects’ can be tracked separately with appropriate experimental designs, e.g., resource addition, canopy or root removal and artificial shading (e.g. Refs.^64,66–68^). Although in the majority of the cases the additivity of the effects is just assumed as valid, it has been occasionally reported that the partial effects were not additive^69^. Theoretical analyses of this issue are rare (but see Ref.^43^), and theoretical mechanistic modelling studies to separate effects in order to test mechanistically whether the net effects can follow a general pattern, such as the SGH predicts, are still lacking.

Here, we set out to study the shapes of the SGH by analyzing the outcomes of a simplified model of a dryland ecosystem, a commonly studied example where facilitation plays a significant role. We use a novel deconstruction of the net effects of plant-plant interactions into partial negative and positive effects along stress gradients. Rather than looking for a general modelling scheme we propose a thorough analysis of one model with three common and representative interactions as a minimal test for the occurrence of the shapes that are compatible with the SGH in a simplified system. We used a resource mechanistic model and focused on drylands. Drylands are a very widespread ecosystems, which cover approximately 41% of the Earth land surface^70^, and are a classical case for the analysis of facilitative interactions among plants^64,71,72^, since they can display high stress levels, due to different types of interaction mechanisms and of stressors (water and grazing being very commonly studied; e.g. Refs.^73,74^). We modelled two plant species that are limited by either water or light^75,76^ and also display a common trade-off between water stress tolerance and optimal growth^77,78^, a combination of traits which is likely to lead to facilitation^53,79–81^. We studied one “resource-mediated” interaction, increase of soil water availability due to vegetation-mediated improved soil properties and water infiltration rates, along a water resource stress gradient, and one “non-resource-mediated” interaction, namely grazing protection, along a grazing stress gradient. In fact, these different ways through which stress acts might be relevant to the SGH shape^82,83^. We did so with a new mechanistic model of plant-resource interactions: based on the standard R* theory^75^, our model is an integration of earlier models of facilitation^78,84^. Many modelling approaches have been applied to explore the validity of the SGH (e.g. graphical models^49^, individual-based explicit space models^85–89^; lottery models^90^; empirical models^91^). The classical R* framework has only occasionally been used explicitly in the SGH context^92,93^. The R* theory provides a resource-competition modeling scheme for species that compete for the same resources^75^ and it has been modified to include interactions with positive effects in two-species systems ^78,84,92–94^. To evaluate interaction effects, we used the unified neighbor indices for intensity and importance^95^, which are the first formally justified and unified set of indices matching the definitions of intensity and importance of neighbor effects.

We addressed the following research questions (RQs), which would test a further applicability of the R* modeling framework in the context of SGH shapes:

RQ1) Which types of qualitatively different SGH shapes can be obtained for the intensity and importance of neighbor effects along rainfall and grazing gradients in drylands?

RQ2) When decomposing the net effects into their partial effects:

a how are partial effects determined by the different interactions at play (e.g. increased infiltration or grazing protection in drylands)?
b do the net effects decompose additively or non-additively into partial effects?

RQ3) How do the separate mechanisms of these interactions determine the SGH shapes?

## Methods

### The full model

We modelled the dynamics of a nurse plant and a protégé plant which compete for two resources, soil water and light, with the protégé plant grazed by herbivores. Our model is based on the R* resource competition theory^75^ and is the combination of two earlier models^78,84^. The model includes schematic and plausible functional responses for the three mechanisms that lead to different interactions between the nurse and the protégé that can have negative (competitive) or positive (facilitative) effects on the protégé. The nurse competes for resources by taking up soil water but can also increase water infiltration in the soil and/or can protect the protégé from being grazed. We considered the two environmental stressors that are directly related to the interactions with positive effects: water availability (related to the infiltration mechanism) and grazing stress (related to grazing protection).

**Figure 1:**
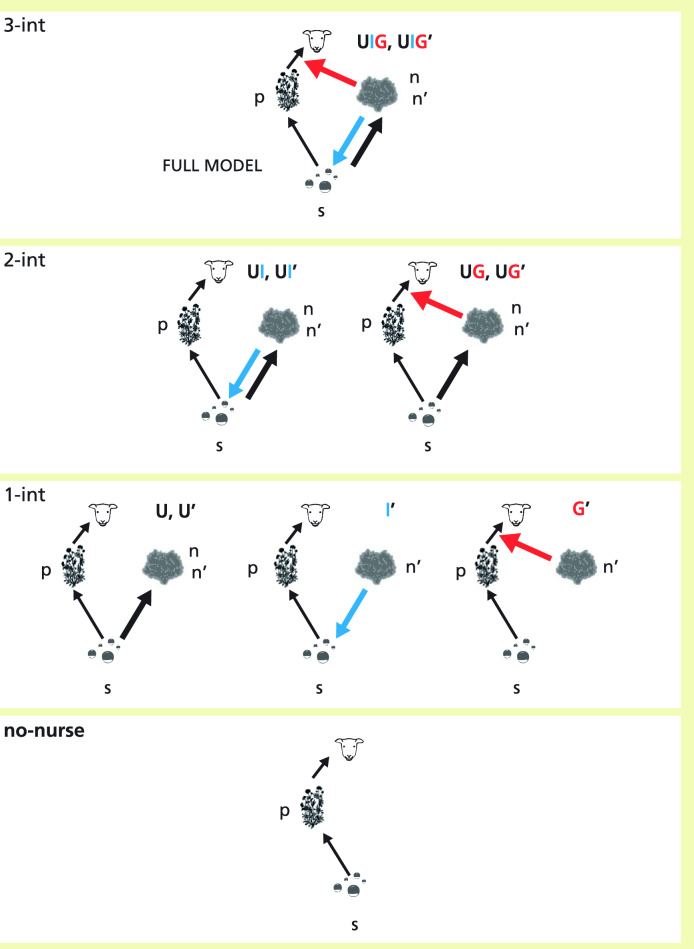
Schematic diagram representing the interactions between protégé and nurse biomass densities (p and n) and soil water s in the full model (Eqs. (1), (2), (3)) and in the int- and sub-models analyzed in the paper, organized in four levels according to the number of plant-plant interactions. Int-models and sub-models are identified by: (i) the plant-plant interactions at play, no-nurse for no mechanisms of interaction (the protégé alone); U for soil water uptake, I for infiltration improvement and G for grazing protection by the nurse; (ii) whether the biomass of the nurse plant is fixed in a certain scenario (sub-models with the apostrophe symbol), or is dynamically determined by resource availability (models with no apostrophe). Light resource is omitted for clarity.

The model consists of three coupled ordinary differential equations, for the dynamics of relative soil water content, *s*, and for the nurse, *n*, and the protégé, *p*, biomass densities. Light availability, *l*, was considered a function of plant biomass since light consumption was instantaneous and non-absorbed light cannot accumulate in the system for later use. The variables of the model and their interactions are illustrated in the top panel of Fig. 1. The equations are the following:

1 $$\frac{ds}{dt}=\frac{1}{\varphi z}\left(r\cdot {\varvec{I}}\left({n}\right)-{\varvec{E}}\left({s}\right)-p\cdot {h}_{p}\cdot {{\varvec{g}}}_{{\varvec{p}}}\left({s}\right)-n\cdot {h}_{n}\cdot {{\varvec{g}}}_{{\varvec{n}}}\left({\varvec{l}}\right)-{\varvec{P}}\left({s}\right)\right)$$

2 $$\frac{dp}{dt}=p\cdot \left({{\varvec{g}}}_{{\varvec{p}}}\left({s}\right)-{m}_{p}-g\cdot \varvec{G}(n,p)\right)$$

3 $$\frac{dn}{dt}=n \cdot \left({{\varvec{g}}}_{{\varvec{n}}}\left(\varvec{l}\right)-{m}_{n}\right)$$

Table 1 describes the variables and the parameters. We refer to Eqs. (1), (2), (3) as the “full model”.

**Table 1:** Model variables and parameters in Eqs. (1), (2) and (3).

	Value	Units	Description
Variables			
s	(0–1)	–	Relative soil water content
n	(0-inf)	g m^−2^	Biomass density nurse
p	(0-inf)	g m^−2^	Biomass density protégé
l	(0–1)	–	Light availability
Parameters			
n’	0–1200 (500)	g m ^−2^	Biomass of the nurse as a parameter
h_n_	3.33	m^2^ mm g^−1^	Amount of water required to produce nurse biomass
h_p_	2.86	m^2^ mm g^−1^	Amount of water required to produce protégé biomass
γ_n_	0.00300	d^−1^	Maximum growth rate, nurse
γ_p_	0.00385	d^−1^	Maximum growth rate, protégé
m_n_	0.0001095	d^−1^	Baseline mortality rate, nurse
m_p_	0.0000913	d^−1^	Baseline mortality rate, protégé
g	(0–0.0055)	d^−1^	Grazing rate, protégé
φ	0.42	–	Porosity
z	200	mm	Soil depth
${{\text{k}}}_{{\text{s}}}$	0.5	mm d^−1^	Saturated hydraulic conductivity
β	0.0002, 0.003 (0–0.01)	m^2^ g^−1^	Infiltrative effect, nurse
i_0_	0.5	–	Infiltration constant bare soil
E_0_	1.61	mm d^−1^	Maximum evaporation at saturation
s_p_	0.072	–	Wilting point, protégé
s_h_	0.08	–	Hygroscopic point
k_p_	0.07201	–	Half saturation constant of the growth rate, protégé
r	(0–1200/365)	mm d^−1^	Rainfall per day
µ	0.2, 0.9 (0–1)	–	Grazing protection
l_0_	1	–	Maximum radiation in the open
l_min_	0.02	–	Minimum light availability, nurse
α_n_	0.060	d^−1^	Light decay rate per unit of biomass, nurse
α_p_	0.035	d^−1^	Light decay rate per unit of biomass, protégé
k_n_	0.22	–	Light absorption half saturation constant, nurse

In Eq. (1), soil water dynamics was determined by a one-layer bucket model representing the relative soil water content (i.e. nondimensional and with values between 0 and 1) for a soil layer of depth *z* and porosity f for both plant types^96,97^. The input term is the infiltration rate, calculated as the product of the average rainfall rate *r* and the soil infiltration capacity ${\varvec{I}}\left({n}\right)=\frac{\beta \cdot n+{i}_{0}}{\beta \cdot n+1}$. The infiltration rate at bare soil is $r\cdot {i}_{0}$ and it increases with nurse density, saturating at *r*; β controls the initial slope of the increment, i.e. the larger the β the higher the nurse improved infiltration (see Fig. S1A). The second term on the r.h.s. (right hand side) of Eq. (1) is the evaporation rate ${\varvec{E}}\left({s}\right)=max\left({E}_{0}\cdot \frac{s-{s}_{h}}{1-{s}_{h}},0\right)$, which is zero below the soil hygroscopic point *s*_*h*_, and linearly increases with soil water content above this threshold. Transpiration of the nurse and the protégé (third and fourth terms on the r.h.s. of Eq. (1)), are proportional to their growth rates, ${{\varvec{g}}}_{{\varvec{n}}}\left(\varvec{l}\right)$ and ${{\varvec{g}}}_{{\varvec{p}}}\left({s}\right)$. The last term is deep percolation rate $\varvec{P}\left(s\right)={k}_{s}\cdot s$, which is proportional to soil water content.

The dynamics of the ‘nurse’ and ‘protégé’ species, Eqs. (2) and (3), included standard Michaelis Menten growth rates and baseline mortalities, *m*_*n*_ and *m*_*p*_. The nurse was limited by light availability, ${{\varvec{g}}}_{{\varvec{n}}}\left(\varvec{l}\right)={\text{max}}\left({\gamma }_{n}\frac{\varvec{l}-{l}_{min}}{\varvec{l}-{l}_{min}+{k}_{n}},0\right)$, and the ‘protégé’ was limited by soil water, ${{\varvec{g}}}_{{\varvec{p}}}\left({s}\right)={\text{max}}\left({\gamma }_{{\text{p}}} \frac{{\text{s}}-{{\text{s}}}_{{\text{p}}}}{{\text{s}}-{{\text{s}}}_{{\text{p}}}+{{\text{k}}}_{{\text{p}}} },0\right)$. In addition, the protégé suffered a grazing mortality (last term on the r.h.s. of Eq. (2)) with a maximum grazing rate, *g*, which is reduced by the nurse plant through a function of the ratio of nurse biomass over protégé biomass, ${\varvec{G}}\left({p},{n}\right)=1-\mu \frac{\frac{n}{p}}{1+\frac{n}{p}}$, to account for size dependence (a small nurse plant cannot protect a larger protégé plant, similarly to Ref.^78^). At the denominator of the grazing term, we used a half saturation constant for grazing protection of 1^78^ so that the protective effect is half its maximum value, *µ*, when both plants have a similar size (i.e. the ratio of *n* over *p* is one): we assumed that, in such case, 0.5·µ of the protégé plant is protected by the nurse (see Fig. S1B,C). Finally, light availability is calculated from plant densities as: ${\varvec{l}}=\frac{{l}_{0}}{1+{\alpha }_{p}\cdot p+{\alpha }_{n}\cdot n}$, thus decreasing with plant density according to a hyperbolic functional form, to model light decay with increasing biomass^98^, where *l*_0_ is maximum radiation in the open, and plant-specific light decay rates per unit of biomass of plant *i* are given by α_i_.

Differently from an earlier study about non-sessile organisms^92^, we considered models where plant species could coexist even in the absence of facilitative interaction along a part of the gradient. This way, we could avoid spurious discontinuities in the SGH shapes that can appear in models where species compete for one resource only^78^. We notice that, although under certain water and light availabilities, the nurse plant would switch to being water-limited and/or the protégé to being light-limited, given our focus on the equilibrium values of scenarios with species coexistence, the shortcut of not including the standard minimum function of a two R-limited growth for each species is valid for the analysis of the research questions.

### Parameter values

We selected parameter values (see Table 1) that are plausible for soil–water balance and vegetation in drylands, generally following^78^ representing a Mediterranean dryland and a common trait combination for two plant species^53^. Thereby, the nurse is less shade resistant and more drought resistant than the protégé^53^ and thus, following the R* theory^75^, the two species coexisted over an extended range of intermediate stress levels (see Fig. S2 and Supplementary S3 for details). The specific parameter values in Table 1 were also selected because they provide direct graphical comparisons of the effects among all the cases along the gradients considered with a single set of values. Additionally, we varied a small subset of the parameters to produce scenarios with different interactions strengths and ranges of stress gradients (see Scenarios section below), while the rest of the parameters was set at the reference values (Table 1). As a sensitivity analysis, we replicated the analysis varying key parameters and the representative values used for the nurse biomass and stress levels (See Supplementary S5).

### Analyses

#### Analysis of the full model and its variants

The full model, Eqs. (1), (2), (3), includes three types of mechanisms of plant-plant interactions (“-int” in the following): competition mediated by light absorption and soil water Uptake (U-int), Infiltration improvement (I-int) and Grazing protection (G-int); see Fig. 1.

To answer RQ1 and identify different SGH shapes along stress gradients, we used four variants of the full model (“int-models” in the following), where different combinations of the interactions were studied by switching off the others. All the int-models included U-int because no plant can survive without consuming resources. They further included either none, one or two facilitative interactions. The int-models were named U, UI, UG and UIG (the full model), depending on the included facilitative interactions. Furthermore, we also used a model for the protégé without the nurse (“no-nurse” in the following), using it as the reference for the characterization of the net neighbor-effects, as in experimental setups.

To answer RQ2, we quantitatively decompose the net effects into partial effects. Typically, in experimental setups, when a net effect is decomposed into partial effects, the same density of the nurse is used in all the treatments, i.e. it does not change as a dynamical function of the interactions considered. To address that, we modified the int-models into “*sub-*models”, by converting the dynamical value of the nurse plant density, *n*, into a parameter, independent of the dynamics, whose value can be freely chosen, *n* = *n*’. We constructed a total of six sub-models, marked with the apostrophe symbol: one for each of the four int-models (U’, UI’, UG’ and UIG’), plus two more including only one facilitative interaction (I’ and G’). See Fig. 1 for a graphical illustration. Differently from the int-models, which simulate real dynamics, the sub-models are mathematical artefacts. Yet, they are useful to isolate the direct effect of one interaction of the nurse on the protégé by removing the indirect effects due to the dynamics of the nurse biomass itself as a function of water availability, Eq. (2). In other words, in the sub-models we broke the feedback loop due to the water competition acting on the nurse density, which in turn affected the biomass of the protégé plant. We also grouped these models by the number of interactions they include: 1-, 2- and 3-int (Fig. 1). The details can be found in Supplementary S2 (Table S2).

#### Stages of the analysis

We first characterized the SGH shapes of the neighbor effects along the stress gradients, using a reverse axis of mean rainfall rate (r in Eq. (1)) for water stress, which increased with decreasing rainfall, and the grazing rate (g in Eq. (2)) for grazing stress. Then, we de-constructed the interactions at play, through an analysis of the sub-models, and used such analysis to interpret the shapes described in the first place.

##### RQ1)

We plotted the SGH shapes of the net effect of interactions in each int-model along the two stress gradients, using two standardized additive neighbor interaction indices to quantify their intensity (NInt_A_) and importance (NImp_A_) ^95^ in terms of the equilibrium biomass densities, calculated by deriving analytical solutions when possible, or otherwise by calculating numerically their values (see Supplementary S3). We did so for suitable ranges of parameter values and stress levels (see sub-section Scenarios below).

##### RQ2)

The sub-models served to separate the dependence of the biomass of the protégé plant in the four int-models on all the elements of the system (water and/or grazing stress, the nurse plant biomass and the plant interactions). The *partial* effect of a single interaction (U-int, I-int or G-int) was quantified as the difference between the performances of the protégé plant in the related 1-int sub-model (U’, I’ or G’) and in the no-nurse model.

We analyzed the protégé density at equilibrium along: (i) each of the two stress gradients, separately, for specific values of the other stress gradient and of the nurse density *n’*; (ii) along an axis given by the nurse biomass, *n’*, at specific values of water and grazing stress (see Scenarios section below). This latter analysis was necessary to fully characterize the system because *n’* is an extra parameter of the sub-models. All the rest was kept fixed. Then, we compared the biomasses of the protégé plant in the 1-int sub-models (U’, I’ and G’) with the values in the no-nurse model along the gradients and linked the observed changes with the properties of the interactions. Additionally, we checked how the nurse density changed along the stress gradients in the int-models, to recover the information the sub-models missed because the nurse biomass was a parameter, disconnected from the dynamics. For simplicity, we referred to the shape of the nurse density in the U model as a proxy for the rest of the int-models (UI, UG and UIG). See Fig. S5 (Supplementary S4.2) for details about the validity of such approximation.

To check whether the partial effects of two or three mechanisms of interaction were additive (RQ2b), we compared the sum of the partial effects obtained with the 1-int sub-models (U’, I’ and G’) along the stress gradients, with their combined effects as obtained by 2 and 3-int sub-models (UI’, UG’ and UIG’). For example, using the two sub-models U’ and I’, we checked whether the partial effects of water uptake (in U’) and infiltration improvement (in I’) along the water stress gradient were equal to their combined effect for UI’ (symbolized as UI’ = ? U’ + I’).

*2.3.1.3 RQ3)* We used the description of the partial effects in the sub-models (U’, I’, G’) from RQ2 to interpret the contribution of each interaction to the SGH shapes in the 4 int-models (U, UI, UG, UIG). We integrated the information obtained in the various analysis performed to answer RQ2, to explain the contribution of each interaction to the shapes of the protégé biomass along the stress gradients.

#### Scenarios

The facilitative interactions G and I were varied at two levels (low and high), by changing the infiltration improvement (β = 0.2 10^–3^, 3 10^–3^ m^2^ g^−1^) for I-int and the maximum grazing protection (µ = 0.2, 0.9) for G-int.

The stress gradients were represented by varying: the rainfall rate *r*, the grazing rate *g* or the fixed nurse biomass *n*’. To avoid the confounding effects of analyzing weak gradients^53^, we explored wide ranges of the stress gradients, so that at high stress level the nurse could outcompete the protégé. Decreasing values of rainfall rate, *r*, were used to account for water stress gradient (from 1250/365 to 0 mm day^−1^). Grazing rate, *g*, was varied from no grazing to 0.0055 day^−1^. When required, we used the following representative values of the parameters: neighbor density, n’ of 500 g m^−2^ (although a sensitivity analysis was performed for values between 50 and 500 g m^−2^); rainfall rate of 600/365 mm day^−1^; grazing pressure of 0.00047 day^−1^.

For the 1 int-models, we did not analyze the SGH shapes along the stress gradients that are unrelated to the facilitative interaction included in the model, i.e. in UI, I-int is unrelated to grazing stress, and in UG, G-int is unrelated to water stress.

## Results

### RQ1)

#### 1-int model, U. (Fig. 2A,B,E,F)

**Figure 2:**
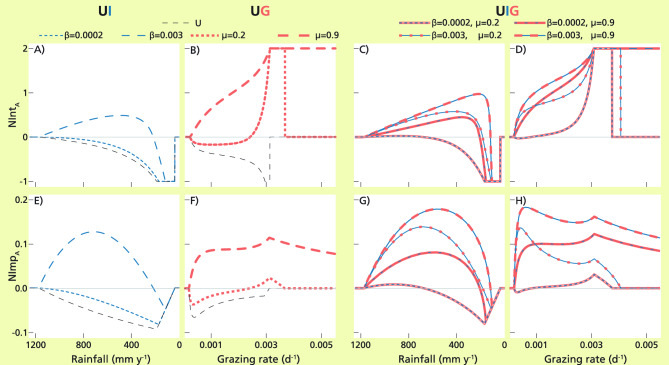
Intensity (as given by NInt_A_, top) and importance (NImp_A_, bottom) shapes along the relevant stress gradients, for models U (black dashed lines; (**A**,**B**,**E**,**F**)), UI (blue dotted and dashed lines; (**A**,**E**)), UG (red dotted and dashed lines; (**B**,**F**)) and UIG (intermittent blue-red lines; (C,D,G,H)). The 1-int U model is a particular case of UI and UG models when the facilitative interactions are null (β = 0 and g = 0). The 2-int models UI and UG are depicted for low and high values of the two interaction strengths (β and µ); 3-int UIG model results are depicted for four combinations of low and high values of β and µ interaction strengths. Values for rainfall rate and grazing rate are r = 600 mm y^−1^ (**B**,**D**,**F**,**H**) and g = 0.00047 d^−1^ (**A**,**C**,**E**,**G**); the rest of the parameter values are as in Table 1. Notice that the y-axis spans the whole range of the intensity index NInt_A_, while the importance NImp_A_ spans only a limited range of values (from − 0.1 to 0.2), as the importance index is much less pronounced in the responses. In the legends, β units are m^2^ g^−1^ and µ is dimensionless.

In general, the values of NInt_A_ and NImp_A_ were increasingly negative with stress until the minimum value was reached at high stress (black dashed lines in Fig. 2A,E along the water stress gradient and in Fig. 2B,F along the grazing stress gradient). The only exception was NImp_A_ as a function of grazing pressure, which reached its minimum value already at low stress values, slightly increasing afterwards (black dashed line in Fig. 2F). The two indices went to zero at high stress, where the protégé was unable to survive, whether with or without the nurse. Transition to the null value was abrupt for intensity, jumping from the maximum values to zero, and more gradual for importance. Intensity reached the index minimum value (NInt_A_ = − 1), while importance had relatively small minimum values (up to − 0.1).

#### 2-int models, UI and UG. (Fig. 2A,B,E,F)

The response of the protégé for UI and UG showed different overall SGH shapes along their related stress gradients (blue lines in Fig. 2A,E for UI along water stress, and red lines in Fig. 2B,F for UG along grazing pressure). For UI and low infiltration effect of the nurse (low β, dotted blue lines in Fig. 2A,E), the shapes of the indices were analogous to those for the U model. For UI and large infiltration effect (large β, dashed blue lines in Fig. 2A,E), the SGH shapes of the indices were hump shaped: NInt_A_ and NImp_A_ had positive values at low water stress, reaching maximum values at intermediate water stress, followed by negative values at higher stress and null values for very high stress. For UG (Fig. 2B,F), large values of the indices occurred at high grazing stress, reaching the maximum value of intensity (NInt_A_ = 2). At low grazing stress, if the grazing protection *µ* was low, indices had negative values, reached a minimum value and displayed an abrupt jump towards maximum values at high pressures (Fig. 2B,F, dotted red lines); if the grazing protection was high, indices were always positive and had a gradual increasing trend with stress. (Fig. 2B,F dashed red lines). The intensity index remained large at very high stress levels, while importance slowly relaxed towards zero after reaching the maximum values.

#### 3-int model, UIG (Fig. 2C,D,G,H)

The SGH shape of the neighbor indices for the full UIG model depended mainly on the type of stress gradient. The shapes of NInt_A_ and NImp_A_ along the water and grazing stress gradients (intermittent blue and red dashed lines in Fig. 2C,G and D,H, respectively) resembled those of UI (Fig. 2A,E) and UG (Fig. 2B,F) along the same gradients. Intensity and importance as a function of water stress were unimodal with large positive values at high stress that decreased into minimum negative values and collapsed where stress was too high for the protégé to survive, with or without the nurse, but importance reached less extreme values. Along the grazing gradient, at low pressure intensity and importance started off either negative or positive, depending on the values of infiltration and grazing protection parameters, they steadily increased with stress until they reached maximum values at high pressures and collapsed where grazing was too high and/or protection too low. Intensity values remained high at very high stress, but importance slowly decreased.

The sensitivity analysis of the parameters revealed that, qualitatively, the shapes obtained were representative of the different effects of the interactions as functions of stress levels (compare Fig. 2 and Figs. S7–S9 in Supplementary S5). The only qualitative change was observed in UG and UIG at low grazing stress, where the biomass of the nurse was very small, either due to low γ_n_ (Fig. S7B,F,D,H, dotted lines) or to low h_n_ (Fig. S8B,F,D,H, dotted lines) and the positive effects had no impact on the net effect, which displayed high negative values.

### RQ2a)

**Figure 3:**
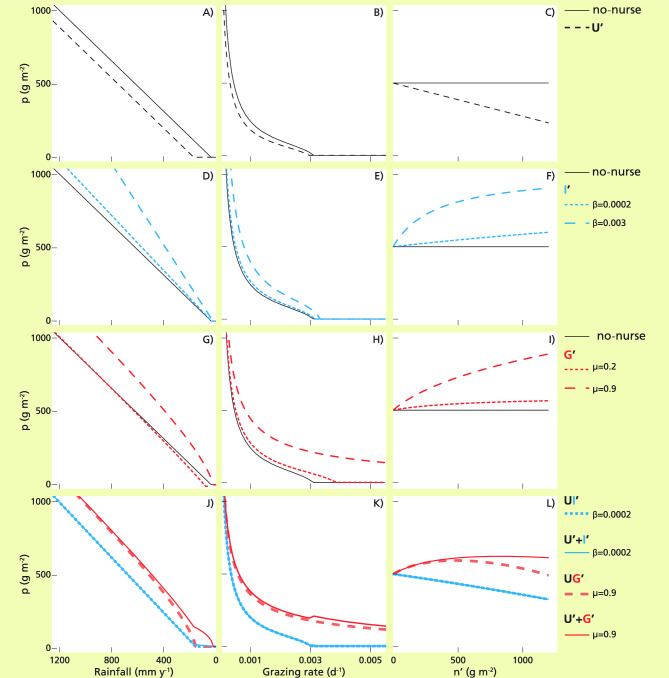
Biomass of the protégé in the 1-int sub-models as a function of water stress (left), grazing stress (middle) and nurse biomass fixed as a parameter, n’, (right). The biomass in the no-nurse model is shown for comparison (black continuous lines, (**A**–**I**)). (**A**–**C**) U’ (black dashed lines), (**D**-**F**) I’ (blue dotted and dashed lines for high and low β interaction intensity) and (**G**-**I**) G’ (red dotted and dashed lines for high and low μ interaction intensity). (**J**–**L**) the sum of the 1-int sub-models (U’ + I’ and U’ + G’, blue and red continuous lines) and the corresponding 2-int sub-models (UI’ and UG’, blue-dotted and red-dashed lines) for the case with low β and high µ interaction intensities. Note that UI’ = U’ + I’ (thus the dashed and continuous lines overlap) while UG’ ≠ U’ + G’. Grazing rate g = 0.00047 d^−1^ (left and right), rainfall rate r = 600 mm y^−1^ (middle and right) and nurse density n’ = 500 g m^−2^ (left and middle); the rest of the parameter values are as in Table 1. See Fig. S4 for the differences between the protégé biomass in the 1-int sub-models and in the no nurse model. In the legends, β units are m^2^ g^−1^ and µ is dimensionless.

**Figure 4:**
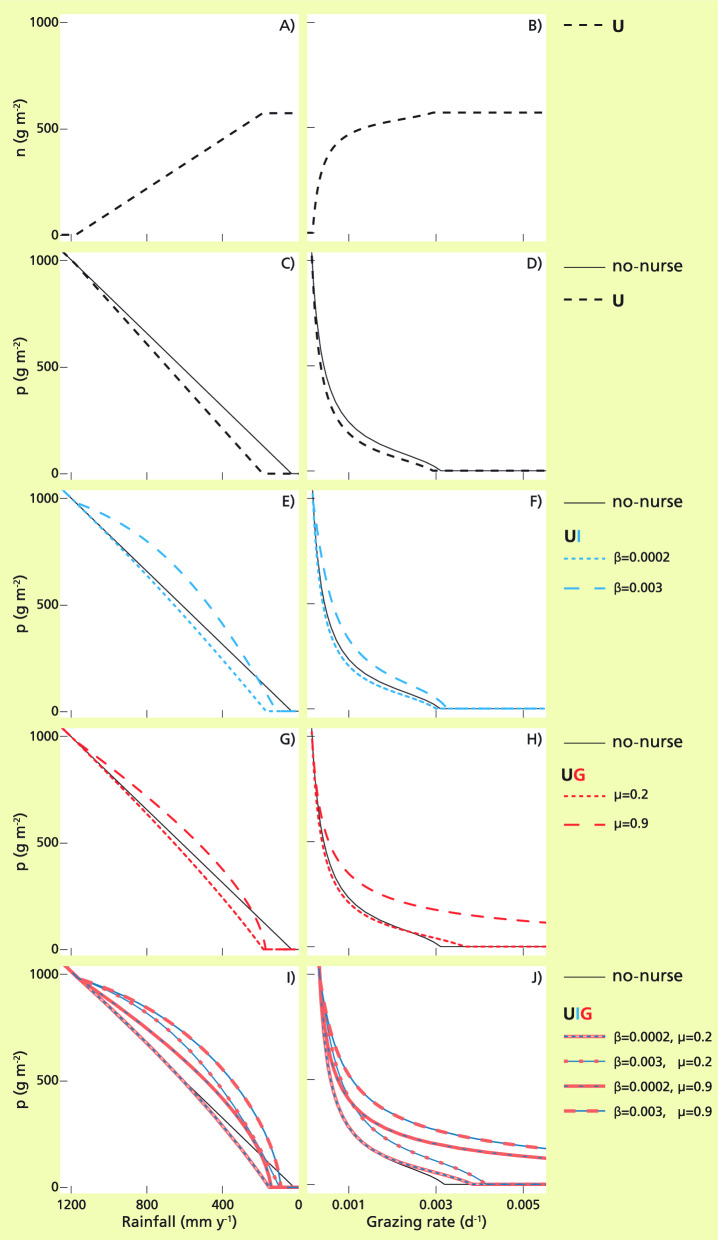
Biomass of the nurse (**A**,**B**) and of the protégé (**C**–**J**) along the water (left) and grazing (right) stress gradients for: no-nurse model (black continuous line; (**C**–**J**));1-int U model (black dashed lines; (**A**–**D**)); 2-int models UI (blue dotted and dashed lines; (**E**,**F**)) and UG (red dotted and dashed lines; (**G**,**H**)), depicted for low and high values of β and µ interaction strengths; 3-int UIG model (intermittent blue-red; (**I**,**J**)), for the four combinations of low and high values, β and µ. Grazing rate g = 0.00047 d^−1^ (left) and rainfall rate r = 600 mm y^−1^ (right); the rest of the parameter values are as in Table 1. See Fig. S5 for differences between the protégé biomass in the 1-int, 2-int and 3-int models and in the no nurse model. In the legends, β units are m^2^ g^−1^ and µ is dimensionless.

The overall shapes of the protégé biomass along the stress gradients in the sub-models with constant nurse (U’, I’ and G’ in Fig. 3A,B,D,E,G,H) and in the int-models with dynamic nurse (U, UI, UG and UIG in Fig. 4) were roughly the same observed for the protégé without the nurse (no-nurse model in Fig. 3A,B,D,E,G,H). Specifically, the biomass of the protégé decreased: (i) linearly with water stress until it became extinct under medium-dry conditions (Figs. 3A,D,G and 4C,E,G,I); (ii) decreased displaying an upward concavity with grazing pressure (Figs. 3B,E,H and 4D,F,H,J). Beyond such general trends, the interactions with the nurse generated deviations from the no-nurse model that we present and analyze in the following sections.

#### The impact of stress on partial effects

The nurse competition in U’ lowers the protégé biomass, with respect to the case without the nurse. The reduction in biomass was the same along the whole water-stress gradient because the nurse water uptake was uncoupled from the water availability (Fig. 3A). Along the grazing gradient (Fig. 3B), the impact of a constant uptake of water from the nurse had a decreasing effect with increasing grazing (Fig. S4B explicitly shows such difference between the protégé biomass in U’ and the no nurse model), showing that the constant uptake of water by the nurse became less relevant for the decreasing density of the protégé when the grazing pressure increased. Contrastingly, in I’ and G’, the nurse presence led to an increase in the biomass of the protégé, but with different trends along each stress gradients. The positive partial effects of I-int diminished along the water-stress gradient (Fig. 3D), because at low precipitation less water was available for the nurse to increase infiltration into the soil. The positive effect of G’ (Fig. 3H and S4H) remained roughly constant along the grazing gradient. Facilitative interactions expanded the realized niche of the protégé plant along the grazing gradient (Fig. 3E,H) but not along the rainfall gradient (Fig. 3D,G). Also, the shape of the biomass of the protégé did not change considerably along the unrelated stress gradient: grazing pressure did not modify much the partial effect of infiltration improvement, except for low stress, (Fig. 3G) and water stress did not change much the effect of grazing protection (Fig. 3E).

#### The impact of the nurse biomass on partial effects

We obtained two distinct shapes of the protégé biomass as a function of the static nurse biomass for 1-int sub-models (U’, I’ and G’). Competition via water uptake affected linearly the biomass of the protégé because water uptake by the nurse increased linearly with its biomass (U’, Fig. 3C). The positive effects increased following convex saturating curves, bounded either by the amount of extra water available for soil infiltration improvement (I’, Fig. 3F) or by how much the herbivory impact could be reduced by grazing protection (G’, Fig. 3I). Thus, per unit of nurse biomass, the negative effect was constant, while the positive effects reduced with higher nurse density. The maximum values of the positive partial effects depended on the interaction strength parameters, i.e. β for the infiltration effect (low and high β, blue lines in Fig. 3F) and the maximum grazing protection, µ (low and high µ, red lines in Fig. 3I).

#### The nurse biomass as a function of stress levels

Because the protégé is increasingly dominant at low drought and/or grazing stress, in U, the nurse biomass increased linearly at low water stress, reaching a maximum constant value at intermediate water stress (Fig. 4A). The biomass of the nurse increased convexly as a function of grazing stress until it converged to a maximum biomass density at high grazing pressures (Fig. 4B). These shapes are complementary to the shapes that the protégé biomass shows along the two gradients (linearly decreasing with water-stress, Fig. 4C, and concave downward with grazing-stress, Fig. 4D) because at equilibrium they contributed to light absorption and water consumption in a similar way (Eqs. (1), (2), (3)). These shapes were the result of stress affecting the competitive ability of the protégé, as the nurse plant had more and more access to light, i.e. its limiting resource, along the stress gradients, as the protégé biomass decreased because of water or grazing stress. In the 2 and 3-int models (UI, UG and UIG), positive effects increased the competitiveness of the protégé, so the increase of the nurse biomass occurred at larger stress values (Fig. S6).

### RQ2b)

The protégé biomass of the 2-int sub-model UI’ was predicted almost exactly by the sum of the corresponding 1-int sub-models, i.e. UI’ = U’ + I’ (Fig. 3J–L, overlapping continuous and dotted blue lines), because both U- and I-int directly affected water. On the contrary, the combined responses of the protégé biomass of UG’ and UIG’ were not the sum of the partial sub-models (UG’ ≠ U’ + G’, UIG’ ≠ U’ + I’ + G’), indicating grazing led to non-additive partial effects (Fig. 3J–L). This is because the impact of the grazing protection is proportional to the protégé biomass exposed to herbivores, which is affected by water competition with the nurse plant, and thus the combined effect of competition and grazing protection cannot be split into independent and additive effects. Water uptake not only reduced soil water content but also prevented the manifestation of the grazing protection, because there was only little biomass of the protégé to be protected. The deviation from additivity was most apparent at high values of the nurse biomass (Fig. 3L, non-overlapping continuous and dashed red lines).

### RQ3)

We identified two general points:

- The replacement of the protégé by the nurse species along the stress gradient was the main driver. The nurse biomass increased mostly linearly, as a function of water stress, and rapidly and then saturating, as a function of grazing stress (Fig. 4A,B);
- Each neighbor index induced specific characteristics in the related SGH shapes. The differences in the protégé biomass values with nurse (int-models) and without (no-nurse model) are non-linearly standardized between maximum and minimum values in NInt_A_ (from − 1 to 2) and NImp_A_ (from − 1 to 2)^95^, inducing vertical ‘compressions’ of the shapes: e.g., a linearly increasing difference in the protégé biomasses becomes a convex SGH shape, while a linearly decreasing one becomes a concave SGH shape. Additionally, NImp_A_ weights the effect of neighbors with respect to the direct effect of stress on the protégé, inducing lower absolute values and more gradual fading towards zero at high values of stress of the importance as compared to intensity shapes. The latter abruptly goes to zero when the limit of the protégé potential niche is reached.

Following are the separate int-models along the stress gradient(s):

- U-model along the water stress gradient (Fig. 2A,E). The linearly increasing effect of competition (see the difference between dashed and continuous lines, in Fig. 4C) is explained by the linear growth of the nurse with water stress gradient, replacing the protégé (Fig. 4A), which caused larger negative effects with increased biomass (linearly increasing differences between no-nurse and U’ in Fig. 3C). The changes in water stress did not have a direct effect (see the fixed difference between no-nurse and U’ in Fig. 3A). NInt_A_ and NImpA responded showing a concave shape (Fig. 2A,E, black dashed lines), with the transitions between the minimum value and zero that were large and abrupt for intensity and small and gradual for importance.
- U-model along the grazing stress gradient (Fig. 2B,F). The effect of U grew rapidly at low grazing stress and decreased slowly with grazing (see the difference between dashed and continuous lines in Fig. 4D; Fig. S5D), driven by the nurse biomass increasing rapidly at low stress and becoming rather constant at high stress (Fig. 4B). Although the grazing stress had larger and larger negative effects because of higher nurse densities (and linearly increasing differences between no-nurse and U’ in Fig. 3C) the nurse biomass had a decreasing effect per unit biomass (Fig. S4B) explaining the higher rate of change of indices, particularly of importance, at low grazing stress. Importance reached its minimum at much lower stress and faded to zero more gradually than intensity, as expected from the indices’ definitions.
- UI model along the water stress gradient (Fig. 2A,E). The effect on the biomass of the protégé increased at low water stress and, after reaching a maximum, decreased, leading to a net negative effect (given by the difference between no-nurse, black line, and UI, blue lines, in Fig. 4E; blue lines in Fig. S5E). This was given by the different effects of U-int (described above) and of I-int along water stress. The effect of the increasing nurse on the protégé along the water stress gradient (Fig. 4A) was modulated by the positive effect of infiltration diminishing with water stress (difference between no-nurse, dashed line, and I’, continuous blue lines, in Fig. 3D), whose effect per unit of nurse biomass saturated with the nurse biomass (Fig. 3F). Thus, the balance tilted towards net positive effects even for very small infiltration increases and towards net negative effects at high stress, while for medium stress the outcomes depended on the infiltration parameter strength (β) (Fig. 2A,E). Facilitation changed into competition more abruptly when the interaction strength (β) was higher, yet at high stress negative effects always dominated. The humped SGH shapes were reinforced by the neighbor indices.
- UG along the grazing stress gradient (Fig. 2B,F). The effect of UG (the difference between no-nurse, black line, and UI, red lines, in Figs. 4H and Fig. S5H) was parameter sensitive at low grazing. The very small partial positive effect was overcome by the negative effect of competition at low grazing stress only for large values of grazing protection (high µ). The positive effect rapidly increased with grazing, leading to net positive effects at intermediate grazing which reached maximum values at high grazing. The net effect was more negative, or less positive, than expected at high nurse densities due to the non-additive combination of the effects of U-int, described above, and the effects of G-int along grazing stress (Fig. 3L). The increase in nurse biomass was fast at low and slow at high grazing stress (Fig. 4B) but was counterbalanced by two factors: a slow increase of the partial positive effect with nurse density (Fig. 3I), giving low protection when nurse density was scarce (differences between no-nurse, black line, and I’, blue lines, in Fig. 3E; blue lines in Fig. S4E); grazing protection not being very relevant on the mortality when grazing pressure was very small, i.e. few grazers to protect the protégé from (difference between no-nurse, black line, and G’, red lines, in Fig. 3H; red lines in Fig. S4H).
- UIG along the water and stress gradients. The SGH shapes of the full model were given by integrating the partial effects of G-int in the already described UI along the water gradient and those of I-int in UG along the grazing gradient. Most of the shapes did not have a significant change when adding a second facilitative interaction (compare in Fig. 2 the shapes in A/C, E/G, B/D, F/H). The infiltration effect contributed more to facilitation at low stress, and grazing protection had a larger impact at high stress (see the comparison of the protégé biomasses obtained with low β and high µ and high β and low µ in Fig. 4I,J). In general, in UIG the net effect of the nurse biomass was positive, and the combination of two partial positive effects overcame the competitive ones, even considering that, due to the non-additivity of the G-int, the net effects were more competitive than the plain sum of partial effects at high nurse biomass. In particular, positive effects had a synergistic effect at the extreme end of the grazing gradient, where the infiltration amelioration was not enough to compensate the stress and competition on the protégé, but that could exert an additional positive effect once the grazing protection was enough to guarantee its survival (Fig. 4J and Fig. S5J). Only under specific conditions, negative net effects were found, e.g. at intermediate water stress and low grazing pressure, where both positive effects due to grazing defense and water infiltration are low.

## Discussion

Our results, using simulations in a mechanistic model for two plant species in dryland, showed the challenges of a qualitatively predicting the SGH shapes of the net effect along stress gradients. Thanks to the modification of the R* modeling framework, we obtained qualitative estimations of how the SGH shapes emerge from the mechanism of interactions showing, for the first time, that partial positive pairwise effects in drylands show more variety of (non-linear) shapes along the stress gradients than negative partial effects, leading to an extensive range of shapes of the net effect (i.e. the SGH shapes). Importantly, for our conceptual simplified model, partial positive and/or negative effects could not generally be summed up to obtain net effects, which rather can be said to emerge from the non-linear dynamics of the mechanisms at play.

Context dependent limits of the determining the SGH shapes have been observed before^4,34^, and the SGH has been partly reformulated to include for example humped shapes and/or waning or collapse of facilitative interactions at high stress^43,58,82^. Our results confirmed that, for drylands, a key factor producing differences in the SGH shape is the underlying nature of the stress^80,82,92^: with “resource stres” the SGH had a marked peak at intermediate levels of water stress (similarly to what proposed by Ref.^49^), while for “"non-resource stres”" intensity of facilitation remained large at high stress but its importance faded gradually (as in e.g. Ref.^55^). Our findings, however, suggest that, at least for drylands, a generic formulation for the SGH shapes does not emerge from the dynamical properties of pairwise interactions. When studying the SGH shapes for models with one or two facilitative interactions, results included an ample variety of shapes, i.e. concave with a maximum value, curves displaying both a minimum and a maximum, or increasing till saturation. We even observed situations with several peaks of facilitation occurring at different levels of stress. This clearly showed that in a rather simple case, i.e. two plants with two facilitative interactions along two independent stress gradients, no generic pattern can be identified for net effects that holds generically along single stress gradients.

While negative partial effects were rather uniform, i.e. linearly increasing with nurse biomass and preserving the shape of the no-nurse case along the stress gradients, positive partial effects displayed various different shapes along the two stress gradients. This partly led to the various shapes of the SGH, and more specifically to the difference between the SGH shapes along the resource and non-resource driven stressors. Along the water stress gradient, the per capita effect of water uptake remained constant (Fig. 3A and Fig. S4A) while the per capita effect of infiltration amelioration decreased (Fig. 3D and Fig. S4D), determining that the maximum facilitation occurred at intermediate water stress for any value of amelioration of infiltration rates β (Fig. 2A,E). Such maximum appeared when the soil water level was low enough for the nurse to increase competitiveness over the protégé, but rainfall was still large enough for the positive effect of infiltration to increase protégé growth. Along the grazing gradient, the per capita effect of water uptake convexly diminished at low grazing rates and attained a rather constant value for intermediate grazing rates (Fig. S4B), while the per capita effect of grazing protection in our model displayed two peaks along the grazing gradient (Fig. 3H and Fig. S4H) determining that the shape changed qualitatively with grazing protection values (µ) and that it may switch between positive and negative values at low grazing stress (Fig. 2B,F). Grazing protection remained large enough for facilitation to reach the maximum value at high grazing stress, because the positive effect over the potentially preventable loss of biomass of the protégé to grazing was still very large. Also in response to changes in the nurse biomass, the partial effects due to the facilitative interactions were markedly different from the partial effects due to resource competition. We observed saturating responses to changes in the nurse biomass (Fig. 3F,I), while competition increased proportionally to it (Fig. 3C). This saturation at high nurse density can be expected in many systems that present facilitation, because the processes behind facilitative interactions tend to have limits that do not depend on the nurse density: in our case, the quantity of rainfall that can be infiltrated or the amount of grazing that can be prevented. This pattern might differ for cases of extreme facilitation, such as ecosystem engineers creating habitats for other species (e.g. in coral reefs), where positive effects could grow proportionally to the nurse density.

Furthermore, our model results showed that in ecosystems and communities where non-linear dynamics are likely to take place, similarly to our dryland case study, different partial positive/negative effects cannot be simply summed up to estimate the net effect, but rather they interact non-linearly in non-trivial ways. Thus, the net interaction effects are not always easy to predict from the partial effects. This has been noticed in some early experiments^27,69^, but experimental studies fail to capture the full combined gradient and its implications had not been explored with mechanistic models and before. In our model, different facilitative interactions could act synergistically rather than additively, for example even producing neutral or positive net effects while separately their effects would be negative at very high water, less than ca. 150 mm y^−1^, or grazing, more than ca. 0.003 d^−1^, stress levels (compare Figs. S5E,G and S5I or S5F,H and S5J for high facilitative interaction strengths parameters, β and µ). Besides, while the partial effects of infiltration improvement and water uptake were additive, the partial effects of grazing protection did not directly sum up to the net effects, which rather was the result of nonlinear interactions. The additivity between water uptake and each of the two facilitative interactions differed because of their different direct or indirect impacts on soil water level (see explanation in Supplementary S3). In general, the non-additivity of partial effects should not be assumed: for example, our model results suggest that when trying to estimate the partial negative effects as the difference between the measured net and the positive partial effects in a system (e.g. Refs.^64,99^), because the net effect is lower than the sum of partial effects, experimenters might end up overestimating the negative partial effects.

Even though it might be difficult to fully and separately turn off the interactions in field observations, appropriately designed manipulative experiments and modelling approaches might reveal that facilitative interactions have larger roles in plant communities than could be detected by traditional pairwise (nurse-protégé) experimental setups. Our mechanistic model showed, in accordance with previous approaches^100^, that facilitative interactions did not necessarily always produce facilitative net effect along the stress gradient even when they reduced the impact of competitive interactions on the biomass of the target, as shown by the results for low infiltration amelioration (dotted blue line in Fig. 2A) or low grazing protection (dotted red line in Fig. 2B). Early experimental analyses of facilitation reported that partial positive effects may be undetected when compensated by negative effects^64^. Although occasionally mentioned afterwards (e.g. Ref.^101^), most experimental studies of facilitative interactions, and their meta-analyses, focus on detecting positive net effect (i.e. only measure the net outcome in terms of survival or growth of the protégé). Even though net positive effects are the most prominent manifestation of facilitative interactions, focusing solely on these excludes a priori the facilitative interactions that do not overcome the effects of competitive interactions. As our results showed, such positive effects might be relevant for plant communities and change along stress levels in ways that might not be predicted if they are not explicitly considered^68^ or even have structural impacts, e.g. plant aggregation^88^. Although facilitation has been considered and used successfully in restoration efforts in drylands^33^, limitations were found in drylands and coastal ecosystem for cases where intraspecific facilitation could not overcome the combinations of stress and disturbance^102,103^. Our novel modelling approach, which separates the positive and negative interactive effects and explores their dependency on biomass (nurse size) and single and combined stress levels, could be applied in future studies to optimize restoration efforts by investigating the optimal size of shrubs given a combination of stress and disturbance.

Our systematic application of neighbor indices in the different versions of the model showed the generality of two properties of intensity and importance that are determined by the definition of the indices^95^, and have been also observed in data analyses. Firstly, they are strongly correlated^55,63^, because both are proportional to the difference of the performances with and without neighbors (Eqs. (S7) and (S8)). Secondly, linear shapes of intensity correspond to humped shapes of importance^47,63^, because of the weighting term, i.e. maximum performance, included in the importance index (Eq. (S8)).

We chose drylands as a classical study case for the analysis of plant-plant facilitation^64,71,72^. A recent broad metanalysis found general support for the SGH, although the effect size was low for plants and did not apply to dryland ecosystems^34^. However, even though our model was designed for drylands, its results might imply that any interaction with a non-linear functional coupling (such as grazing had in our model) would lead the SGH to fail. The failure to detect the SGH experimentally can also be due to other known problems such as the length of the gradient studied^53,79,80,82^: while this limitation can be easily overcome in modelling approaches, a too short gradient in experiments might lead to failure of detection of some SGH shapes.

We acknowledge that our results have limitations. Our modelling scheme, following most experimental approaches and field observations, was designed to study facilitation represented pairwise interactions isolated from more complex community interactions, whose aggregated dynamics might show emergent properties elusive to our approach. Conceptual models aim to grasp the essential patterns of complex systems with a few key processes that are expected to qualitatively govern the dynamics. In this particular case, other mechanisms might play a role, such as e.g. intraspecific physiological acclimation to stresses or idiosyncratic interspecific interactions, but cannot be incorporated in our model. Furthermore, there might be turnover of a variety of mechanisms, each with different strengths, acting along a large environmental gradient. As a result, various SGH shapes or patterns of shapes might emerge as the aggregated effect of this diversity. This combination of different temporal ranges and trends, both increasing and decreasing along the stress gradients, could lead to various shapes that cannot be predicted a priori. An analysis of such aggregated net effect could combine our methodological approach with a statistical treatment of interactions traits along the gradient.

Secondly, the results were derived for a specific but general trade-off relationship between competition and stress tolerance of the protégé and the nurse, which guarantees the coexistence of the two species in the model along a part of the gradient, and parameter values were kept constant along stress gradients. However, this trade-off is relevant and common (e.g. Refs.^53,75–77,79^).

The mechanistic approach to study facilitation in drylands that we implemented showed the importance of the mechanisms at play and how they contribute to the observed net effect, which we speculate can be extrapolated to other ecosystems with positive interactions of differing nature. Given the importance of non-linear dynamics, a generic pattern for the net effect along all stress gradients cannot be expected, which highlights the difficulties of determining general SGH shape. We maintain that studying the mechanisms at play and decomposing the system into sub-models to study them separately is a rather intuitive analysis and could contribute to extend the debate around the SGH shapes, clarifying the role of different factors, providing sharper meaning to unprecise concepts and/or facilitating the formulation of other models (e.g. proposing more general interaction parameters in Lotka-Volterra models^103^ as a function of environmental gradients that include facilitative and competitive interactions). Finally, we consider that our approach could inspire specific experimental designs by aiming to deconstruct mechanism at play and separate their individual and joint effects and advance the analysis for the role of facilitative interactions in more complex cases, such as simultaneous stress gradients or larger communities.

### Supplementary Information

Supplementary Information.

## Data Availability

Data sharing is not applicable to this article as no new data were created or analyzed in this study.
