# Supplementary material for: Facilitation and competition deconstructed: a mechanistic modelling approach to the stress gradient hypothesis applied to drylands
Source: Sci Rep. 2024 Jan 25;14:2205. doi: 10.1038/s41598-024-52447-z (PMC10810957; doi:10.1038/s41598-024-52447-z)
Supplement: Supplementary file 1 — Supplementary Information. [file 41598_2024_52447_MOESM1_ESM.pdf]

SUPPLEMENTARY INFORMATION

Facilitation and competition deconstructed: A mechanistic modelling approach to the Stress Gradient Hypothesis applied to drylands

Rubén Díaz-Sierra[1,2,3], Max Rietkerk[2], Mart Verwijmeren[2,4] and Mara Baudena[5,6,2,3]

[1]Mathematical and Fluid Physics Department, Faculty of Sciences, Universidad Nacional de Educación a Distancia, UNED, Madrid 28040, Spain

[2]Copernicus Institute of Sustainable Development, Environmental Science Group, Utrecht University

[3]Centre for Complex Systems Studies, 4th floor Minnaert building, Leuvenlaan 4, Utrecht, The Netherlands

[4]National Institute for Public Health and the Environment (RIVM), Bilthoven, the Netherlands

[5] National Research Council of Italy, Institute of Atmospheric Sciences and Climate (CNR-ISAC), Corso Fiume 4 10133 Torino, Italy

[6] National Biodiversity Future Center, - Palermo 90133, Italy.

Index

S1. Definitions, main concepts and positive functional responses. 3

Table S1 3

Figure S1 4

S2. Modifications of the equations of the full model in the int- and sub-models. 5

Table S2. 5

S3. Analysis of int- and sub-models 6

Table S3. 12

Table S4 13

Figure S2. 14

Figure S3 15

S3. Additivity of interactions 16

S4. Supplementary figures 17

S4.1 Difference in the biomass of the protégé between the no-nurse model and the int- or sub-models 17

Figure S4 18

Figure S5. 19

S4.2 Nurse biomass in all the int-models 20

Figure S6. 21

S5. Sensitivity analysis. 22

Figure S7. 23

Figure S8. 24

Figure S9 25

S1. Definitions, main concepts and positive functional responses.

Table S1. Definitions of the main concepts as used in the text (following e.g. Callaway, 2007; Cabal et al., 2020).

	**Definition**
**Interaction**	A biophysical process by which a (nurse) species affects the environment in a way that impacts the performance of a neighbour (protégé) species. Being a mechanism, its effect does not have an associated sign *per se,* and its contribution to the net effect depends on the rest of the interactions and the environmental conditions. However, it is common to refer to positive/negative interactions when their contribution to the net effect has a fixed positive/negative sign in a particular context and/or set of environmental conditions.
**Net effect**	A measurement of the net outcome of all the interactions of a (nurse) species on the performance (biomass in our case) of a neighbouring (protégé) species in a particular system (interactions and environmental conditions).
**Competition**	A negative net effect.
**Facilitation**	A positive net effect.
**Partial effect**	The outcome of an interaction on the performance of a neighboring (protégé) species when the rest of the interactions are switched off and the (nurse) species is kept at constant density. Partial effects can be positive, null or negative. They can also switch between positive and negative depending on the environmental conditions.

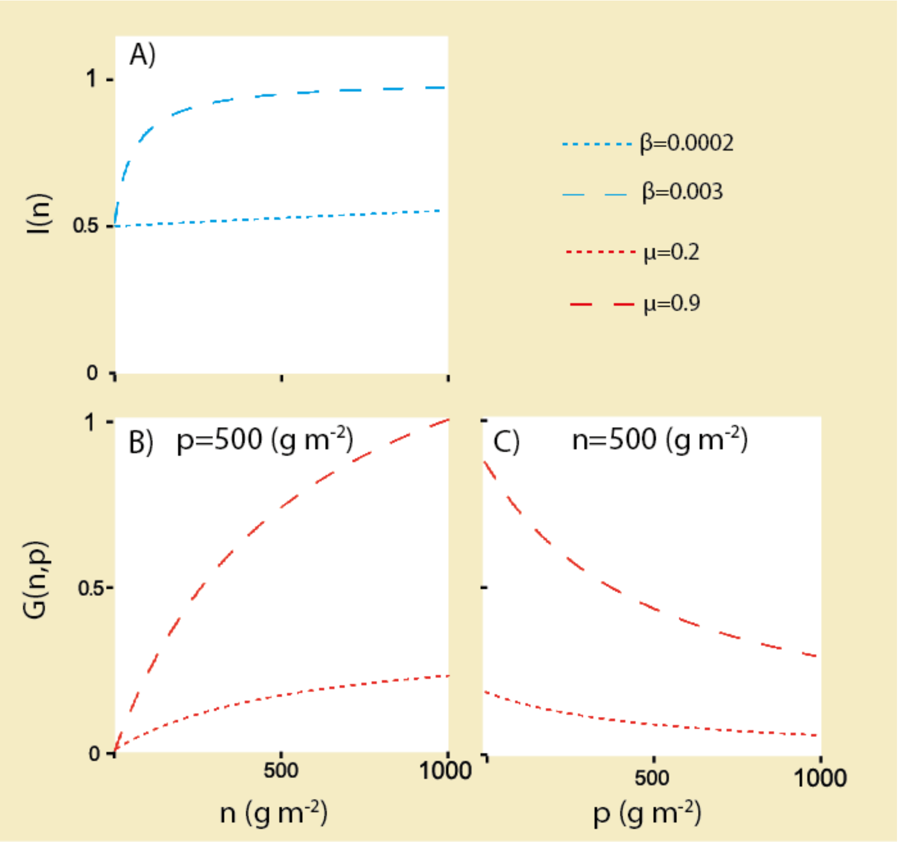

Figure S1. Functional responses that represent the positive interactions in the full model, Eqs. (1)-(2). Soil infiltration capacity as a function of nurse biomass (A) and grazing protection as a function of: the nurse biomass at a fixed value of the protégé biomass (B), and the protégé biomass at a fixed value of the nurse biomass (C). The parameter values are as in Table 1.

S2. Modifications of the equations of the full model in the int- and sub-models.

The biomasses of the plants along stress gradients, as reported in the Results section, (figs 3-4), are the stable equilibria of the int- and sub-models, derived from Eqs.. (1)-(4) (Fig. 1). In Table S2 we explain in detail how these Eqs. were modified or removed to obtain the different int- and sub-models.

Table S2. Equations of the full model (Eqs. (1)-(4); columns) included without modification (tick), discarded (cross) or modified (see the expressions of the substitutions made in the original equations) in each of the model used in the text and Appendices.

	**Eq. (1)**	**Eq. (2)**	**Eq. (3)**	**Eq. (4)**
**no-nurse**	$n = 0$	$\mu = 0$	×	$n = 0$
**no-protégé**	$p = 0$	×	✓	$p = 0$
**U**	$\beta = 0$	$\mu = 0$	✓	✓
**UI**	✓	$\mu = 0$	✓	✓
**UG**	$\beta = 0$	✓	✓	✓
**UIG**	✓	✓	✓	✓
**U’**	$n = n', \beta = 0,$ $\gamma_n \frac{l-l_{min}}{l+k_n} = m_n$	$\mu = 0$	×	$n = n'$
**I’**	$n = n'$	$\mu = 0$	×	$n = n'$
**G’**	$n = n', \beta = 0$	$n = n'$	×	$n = n'$
**UI’**	$n = n',$ $\gamma_n \frac{l-l_{min}}{l+k_n} = m_n$	$\mu = 0$	×	$n = n'$
**UG’**	$n = n', \beta = 0$	$n = n'$	×	$n = n'$
**UIG’**	$n = n',$ $\gamma_n \frac{l-l_{min}}{l+k_n} = m_n$	$n = n'$	×	$n = n'$

S3. Analysis of int- and sub-models.

The equilibrium solutions are reported in Tables S3 (int-models) and S4 (sub-models). The values of the equilibrium protégé biomass in models without the G interaction could be calculated analytically (no-nurse, U, U', I', UI and UI'), while for the models including G-int (G', UG, UG', UIG and UIG') were found numerically (solutions of polynomials of order 3 or 4). We performed numerical and analytical calculations of the equilibria and their stability using Maple™ software (Maple 2021). In the following text, the values of all the state variables at the stable equilibrium are denoted by a tilde above the variable plus the name of the model in the subscript, e.g. $\tilde{s}_{UI}$ for the soil water content at the stable equilibrium of model UI. We do not use this notation in the figures, where the state variables at equilibrium are shown without tilde, as in the main.

We needed to identify a large region of coexistence of protégé and nurse in the parameter space of the water and grazing stress gradients, to perform the analysis in the main. To do so, we analyzed the solutions of the int-models to determine where one or both species existed. We derived the equations of the potential niches of each species in monoculture, and of the realized niches of the species when grown together, which contained regions of coexistence, i.e. where the two species are both present, and regions of species dominance, i.e. where one species always outcompetes the other (Fig. S2). The potential niche of the protégé species was determined from where positive steady states of the protégé biomass in the no-nurse model existed in the stress gradients parameter space (Fig. S2A, dark grey "p" region). Similarly, the potential niche of the nurse was calculated using a model without the protégé ("no-protégé" model, see Table S2; stable and feasible solutions can be seen in Fig. S2B, light grey "n" region). Note that this model was not introduced in the main text. Whether the nurse and/or the protégé had positive values at the stable equilibria determined the following regions: coexistence (Fig. S2B-D, colored

“p+n” regions: U black region in b and dark blue/red/violet in C/D/E; UI, blue region in C; UG, red region in D; UIG, violet region in E); protégé dominance (Fig. S2 C-D, light grey “p” region); protégé dominance (Fig. S2 C-D, light grey “p” region).

In the following we describe in detail how we derived the solutions of the models used:

- **No-nurse model** (Fig. S2A). Solving Eq. (2) for the soil water content yielded the soil water critical value for the survival of the protégé (Diaz-Sierra et al 2010), $\tilde{s}_{no-nurse}$ (Table S3). Substituting $s = \tilde{s}_{no-nurse}$ and n = p = 0 in Eq. (1) we obtained the rainfall rate minimum values for the transition threshold between bare soil and a monospecific stand of the protégé species (Diaz Sierra et al 2010) (in the following, $r^*_{p/0}$):

$$r^*_{p/0} = \frac{1}{i_0}\left(\frac{\tilde{s}_{no-nurse} - s_h}{1 - s_h} - k_s\right), \qquad \text{(S1)}$$

  i.e. the potential niche of the protégé species (Fig. S2A, dark grey) where the protégé has a positive solution, that is always stable. Substituting $p = \tilde{p}_{no-nurse}$ and n = 0 in Eq. (4) gave $\tilde{l}_{no-nurse}$ (Table S3).
- **No-protégé model** (Fig. S2B-E). Without the protégé the biomass of the nurse was constant along the gradient, because it was not directly affected by the water or grazing stress. The nurse biomass was only limited by its light critical value for survival (Díaz-Sierra et al 2010), $\tilde{l}_{no-prot}$ (Table S3), given by solving Eq. (3). Substituting $l = \tilde{l}_{no-protege}$ and p = 0 in Eq. (4) yielded the biomass of the nurse, $\tilde{n}_{no-protege}$ (Table S3), which, when used in Eq. (1), determined the soil water content at equilibrium, $s_{no-protege}$. Rainfall rates where soil water content was positive defined the threshold between bare soil and monoculture of the nurse

species (in the following, $r^*_{n/0}$), i.e. the potential niche of the nurse (Fig. S2B, light grey region):

$$r^*_{n/0} = \frac{\beta \cdot n_S + 1}{\beta \cdot n_S + i_0}\left(\frac{l_0 - \tilde{l}_{no-protege}}{\alpha_n \, \tilde{l}_{no-protege}} \, h_n \, m_n - E_0 \frac{s_h}{1 - s_h}\right) \tag{S2}$$

- **Full and int-models**. The protégé and nurse species coexisted in U, UI, UG and UIG when the following conditions were met (Tilman, 1982):

  i) Each species can invade the steady state of a monoculture of the other species. We derived the two conditions entailed:

  i.1) The nurse can invade the protégé if $\tilde{l}_{no-protege} < \tilde{l}_{no-nurse}$, i.e. the protégé does not control the nurse via shading. Solving for the protégé biomass in $\tilde{l}_{no-protege} = \tilde{l}_{no-nurse}$ gave the threshold, for the full and the int-models, between dominance of the protégé species (Fig. S2B-D, dark grey region) and species coexistence (Fig. S2D/C/D/E, black/blue/red/violet regions) (in the following, $r^*_{p/p+n}$):

$$r^*_{p/p+n}(g) = \frac{1}{i_0}\left(\frac{h_p(m_p + g)}{\alpha_p}\left(\frac{\gamma_n(l_0 - l_{min}) - m_n(l_0 + k_n)}{\gamma_n l_{min} + m_n \, k_n}\right) + k_s \, \frac{\gamma_p s_p + k_p(m_p + g)}{\gamma_p - m_p - g} + \frac{E\left(\frac{\gamma_p s_p + k_p(m_p + g)}{\gamma_p - m_p - g} - s_h\right)}{1 - s_h}\right) \tag{S3}$$

  i.2) The protégé can invade the nurse if $\tilde{s}_{no-protege} > \tilde{s}_{no-nurse}$, i.e. the nurse does not control the protégé via soil water depletion. In the region that delimits

nurse dominance, the grazing protection term was simplified to its maximum value:

$$g\left(1-\mu\frac{\frac{n}{p}}{1+\frac{n}{p}}\right)\xrightarrow[p\to 0]{} g(1-\mu), \qquad \text{(S4)}$$

which allowed the analytical calculation of the biomass of the protégé (Eqs. (1) and (3)) along the threshold between dominance of the nurse species (Fig. S2B-D, light grey regions) and species coexistence (Fig. S2B/C/D/E, black/blue/red/violet regions) (in the following, $r^*_{p+n/n}$) in the full and the int-models:

$$r_{p+n/n}=\frac{\beta\cdot\frac{l_0-l_n^*}{\alpha_n\, l_n^*}+1}{\beta\cdot\frac{l_0-l_n^*}{\alpha_n\, l_n^*}+i_0}\left(\left(\frac{E_0}{1-s_h}+k_s\right)\left(\frac{\gamma_p s_p+k_p\big(m_p+g\cdot(1-\mu)\big)}{\gamma_p-m_p-g\cdot(1-\mu)}\right)\right.$$
$$\left.+\frac{l_0-l_n^*}{\alpha_n\, l_n^*}\,h_n\, m_n-E_0\frac{s_h}{1-s_h}\right) \qquad \text{(S5)}$$

ii) intraspecific competition is higher than interspecific competition. For our models, such condition translated as the ratio between water consumption and light absorption being higher for the nurse than for the protégé. Mathematically, this condition was given by the sign of the Jacobian's determinant at the steady state (Diaz-Sierra et al 2010, Eq. (C6)):

$$\frac{\alpha_n}{h_n\, m_n}>\frac{\alpha_p}{h_p\,\left(m_p+g\right)} \qquad \text{(S6)}$$

Notice that the parameter values in Table 1 fulfill Eq. (S6) for any value of grazing rate.

Fig. S3 is a replica of Fig. 4 (A-H) incorporating the notation used in this Appendix. Notice that the expressions of the regions $r^*_{p/0}$ (Eq. (S1)), $r^*_{p/p+n}$ (Eq. (S3)) and $r^*_{p+n/n}$ (Eq. (S5)) can also be solved for g as a function of r, to obtain $g^*_{p/0}$, $g^*_{p/p+n}$ and $g^*_{p+n/n}$.

- **Sub-models**. To solve these, we removed Eq. (3) in the sub-models that contained U-int (i.e. U', UI' and UIG'), and the term $\gamma_n \frac{l-l_{min}}{l+k_n}$ in Eq. (1) was substituted by m_n, which accounted for the soil water uptake of a nurse plant with constant *n*' biomass. Then, we substituted *n* by *n'* in Eqs. (1), (2) and (4) and solved them as we did in the previous paragraphs for the full and the int-models. Table S4 displays the expressions of the biomass of the protégé. There, we reported these values as the biomass of the no-nurse model plus/minus the expressions that represent partial positive effects (blue boxed text) and negative effects (bold black boxed text). Comparing $\tilde{p}_{U'}$ and $\tilde{p}_{I'}$ with $\tilde{p}_{UI'}$ we notice the additivity of U-int and I-int (UI' = U' + I'). An equivalent visualization of the non-additivity of U-int and G-int (UG' ≠ U' + G') is not available because of the high order of the polynomials involved in the solution of $\tilde{p}_{U'}$, $\tilde{p}_{G'}$ and $\tilde{p}_{UG'}$, although the non-linear effects are apparent by the same reason.

The additively symmetric intensity (NInt$_A$) and importance (NImp$_A$) indices are defined as (Díaz-Sierra et al. 2017):

$$NInt_A = 2\frac{\tilde{p}_{+\mathrm{n}} - \tilde{p}_{\mathrm{no-nurse}}}{\tilde{p}_{\mathrm{no-nurse}} + |\tilde{p}_{+\mathrm{n}} - \tilde{p}_{\mathrm{no-nurse}}|} \quad (S7)$$

$$NImp_A = 2\frac{\tilde{p}_{+\mathrm{n}} - \tilde{p}_{\mathrm{no-nurse}}}{2\cdot Mp_{\mathrm{no-nurse}} - \tilde{p}_{\mathrm{no-nurse}} + |\tilde{p}_{+\mathrm{n}} - \tilde{p}_{\mathrm{no-nurse}}|} \quad \text{(S8)}$$

where $\tilde{p}_{+\mathrm{n}}$ is the protégé plant density with a neighbor (i.e. $\tilde{p}_U$, $\tilde{p}_{UI}$, $\tilde{p}_{UG}$ or $\tilde{p}_{UIG}$ obtained as the equilibrium biomass in one of the int-models; Table S3), $\tilde{p}_{\mathrm{no-nurse}}$ is the protégé biomass without the nurse (obtained as the equilibrium value of the no-nurse model; Table S3,), and $Mp_{\mathrm{no-nurse}}$ is the maximum value of the equilibrium biomass of the protégé species attained without a neighboring nurse at any point along the two gradients (typically, at maximum precipitation and minimum grazing rate, in the no-nurse model).

Table S3. Analytical solutions of all the state variables in the no-nurse, no-protégé and U model, of soil water content in the UI model and of light availability in all the models. For the rest of the cases, the order of the polynomials that were solved (analytically for UI and numerically for UG and UIG) are indicated. Expressions derived for UI (analytical solutions of the quadratic equations) are not displayed for simplicity.

	$\tilde{p}$	$\tilde{n}$	$\tilde{s}$	$\tilde{l}$
**no-nurse Fig. 3 A-I, 4B-C, black continuous lines**	$\tilde{p}_{no-nurse} = \frac{1}{h_p(m_p+g)} \left(r\, i_0 - \frac{E(\tilde{s}_{no-nurse} - s_h)}{1-s_h} - k_s\, \tilde{s}_{no-nurse} \right)$	-	$\tilde{s}_{no-nurse} = \frac{\gamma_p s_p + k_p(m_p+g)}{\gamma_p - m_p - g}$	$\tilde{l}_{no-nurse} = \frac{l_0}{1+\alpha_p \tilde{p}_{no-nurse}}$
**no-protege**	-	$\tilde{n}_{no-protege} = \frac{l_0 - l_n^*}{\alpha_n\, l_n^*}$	$s_{no-protege} = \frac{1}{\frac{E_0}{1-s_h} + k_s} \left(r \cdot \frac{\beta \cdot \tilde{n}_{no-prot} + i_0}{\beta \cdot \tilde{n}_{no-prot} + 1} - \tilde{n}_{no-prot}\, h_n\, m_n + E_0 \frac{s_h}{1-s_h} \right)$	$\tilde{l}_{no-protege} = \frac{\gamma_n l_{min} + m_n\, k_n}{\gamma_n - m_n}$
**U Fig. 4A-D, black dashed lines**	$\tilde{p}_U = \frac{1}{\alpha_n h_p m_p - \alpha_p h_n m_n} \left(\left(r \cdot i_0 - E_0 \cdot \frac{\tilde{s}_{no-nurse} - s_h}{1-s_h} - k_s \cdot \tilde{s}_{no-nurse} \right) \alpha_n - \left(\frac{l_0}{\tilde{l}_{no-prot}} - 1 \right) m_n\, h_n \right)$	$\tilde{n}_U = \frac{1}{\alpha_n h_p m_p - \alpha_p h_n m_n} \left(\left(\frac{l_0}{\tilde{l}_{no-prot}} - 1 \right) m_p\, h_p - \left(r \cdot i_0 - E_0 \frac{\tilde{s}_{no-nurse} - s_h}{1-s_h} - k_s \tilde{s}_{no-nurse} \right) \alpha_p \right)$	$\tilde{s}_U = \tilde{s}_{no-nurse}$	$\tilde{l}_U = \tilde{l}_{no-prot}$
**UI Fig. 4E-F, blue lines**	$\tilde{p}_{UI}$	$\tilde{n}_{UI}$	$\tilde{s}_{UI} = \tilde{s}_{no-nurse}$	$\tilde{l}_{UI} = \tilde{l}_{no-prot}$
	2nd order polynomials (Analytical)			
**UG Fig. 4G-H, red lines**	$\tilde{p}_{UG}$	$\tilde{n}_{UG}$	$\tilde{s}_{UG}$	$\tilde{l}_{UG} = \tilde{l}_{no-prot}$
	3th order polynomials (Numerical)			
**UIG Fig. 4E-F, red-blue lines**	$\tilde{p}_{UIG}$	$\tilde{n}_{UIG}$	$\tilde{s}_{UIG}$	$\tilde{l}_{UIG} = \tilde{l}_{no-prot}$
	4th order polynomials (Numerical)			

Table S4. Analytical solutions of all the state variables in the U' and I' models and of light availability in all the models. For the rest of the cases, the order of the polynomials that were solved (numerically for G', UG' and UIG') are indicated. Expressions of the partial effects of the interactions in U', I' and UI' are highlighted (boxes): negative partial effect of U-int (black bold) and positive partial effect of I-int (blue). These expressions of the partial effects show the additivity of U' and I' in UI' and UIG' while the non-additivity of U' and G' precluded similar separation in UG' and UIG'.

	$\tilde{p}$	$\tilde{n}$	$\tilde{s}$	$\tilde{l}$
**U'**<br>**Fig. 3A-C dashed lines**	$\tilde{p}_{U'} = \tilde{p}_{no-nurse} - \boxed{\dfrac{\boldsymbol{n'h_n m_n}}{\boldsymbol{h_p(m_p+g)}}}$	n'	$\tilde{s}_{U'} = \tilde{s}_{no-nurse}$	$\tilde{l}_{U'}(*)$
**I'**<br>**Fig. 3D-F blue lines**	$\tilde{p}_{I'} = \tilde{p}_{no-nurse} + \boxed{\dfrac{1}{h_p(m_p+g)}\left(r\dfrac{\beta\, n'\,(i_0-1)}{\beta\, n'+1}\right)}$	n'	$\tilde{s}_{I'} = \tilde{s}_{no-nurse}$	$\tilde{l}_{I'}(*)$
**G'**<br>**Fig. 3G-I red lines**	$\tilde{p}_{G'}$<br>3th order polynomials (Numerical)	n'	$\tilde{s}_{G'}(< \tilde{s}_{no-nurse})$	$\tilde{l}_{U'}(*)$
**UI'**<br>**Fig. 3J-B**<br>**blue dotted lines**	$\tilde{p}_{UI'} = \tilde{p}_{no-nurse} + \dfrac{1}{h_p(m_p+g)}\left(r\dfrac{\beta\, n'\,(i_0-1)}{\beta\, n'+1}\right) - \dfrac{\boldsymbol{n'h_n m_n}}{\boldsymbol{h_p(m_p+g)}}$	n'	$\tilde{s}_{UI'} = \tilde{s}_{no-nurse}$	$\tilde{l}_{UI'}(*)$
**UG'**<br>**Fig. 3J-B**<br>**red dashed lines**	$\tilde{p}_{UG'}$<br>3th order polynomials (Numerical)	n'	$\tilde{s}_{UG'}$<br>$(< \tilde{s}_{G'})$	$\tilde{l}_{UG'}(*)$
**UIG'**	$\tilde{p}_{UIG'}$<br>3th order polynomials (Numerical)	n'	$\tilde{s}_{UIG'}$	$\tilde{l}_{UIG'}(*)$

$$(*)\ \tilde{l}_{model'} = \frac{l_0}{1+\alpha_p\,\tilde{p}_{model'}+\alpha_n\, n'}$$

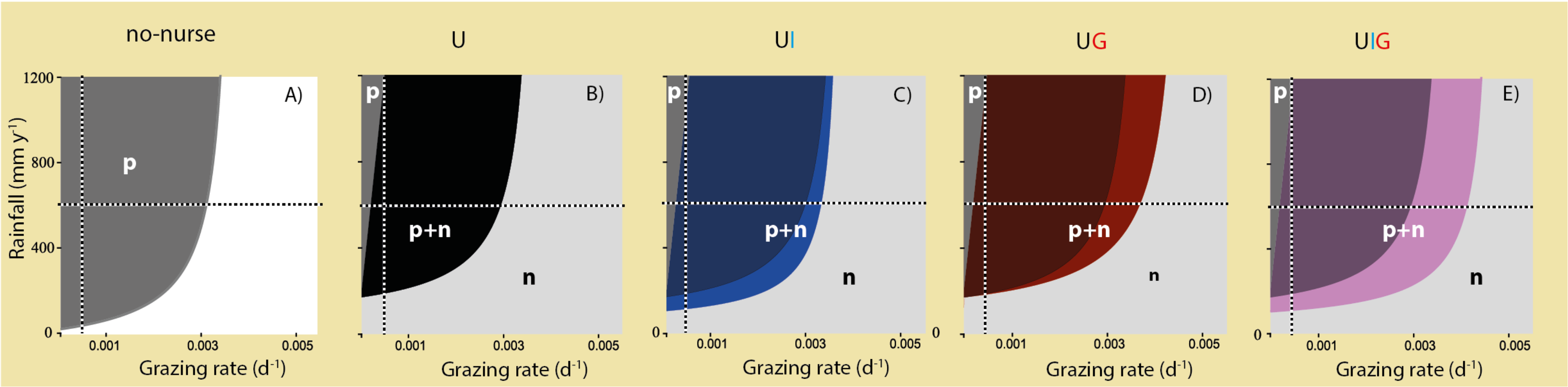

Figure S2. Regions of existence (i.e. feasible positive equilibria) of the protégé and/or the nurse species for the (A) no-nurse, int- (B): U, (C): UI and (D): UG) and (E) full model, in the parameter space of the stress gradients. (A-E) region of existence of the protégé (dark grey) in the no-nurse model; (B-E) regions of: nurse only (light grey) and coexistence ((B), black; (C), blue; (D), red; (E) violet). In (C-E)_the region of coexistence in the U model is shown in darker colours, for comparison with the 2 and 3-int models. Horizontal and vertical black&white dotted lines are the representative values of the stress gradients used in Fig. 4, r=600 mm y^{-1} (Fig. 4B,D,F,H,J) and g=0.00047 d^{-1} (Fig. 4A,C,E,G,I), for describing the biomasses of the nurse (Fig. 4A,B) and the protégé (Fig. 4C-I) in the int-models along the relevant stress gradients. β=0.003 m^2 g^{-1} in (C,E) and μ=0.2 in (D,E), the rest of the parameter values are as in Table 1.

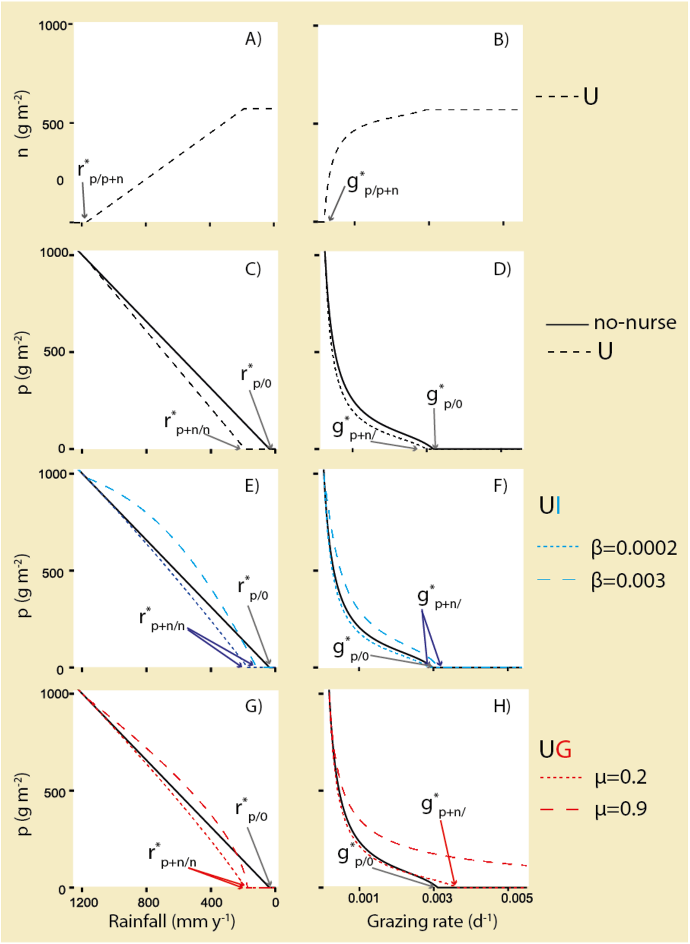

Figure S3. Replica of Fig. 4 A-H, showing the location of state transition points between: (A-B) protégé dominance and coexistence, $r^*_{n/0}$ (Eq. (S2)) and $g^*_{n/0}$; (C-H) protégé dominance and coexistence, $r^*_{p+n/n}$ (Eq. (S5)) and $g^*_{p+n/n}$; (C-H) Limits of the protégé species survival, $r^*_{p/0}$ (Eq. (S1)) and $g^*_{p/0}$. Biomass of the nurse (A-B) and of the protégé (C-H) along the water (left) and grazing (right) stress gradients for: no-nurse model (black continuous line; C-H);1-int U model (black dashed lines; A-D); 2-int models UI (blue dotted and dashed lines; E-F) and UG (red dotted and dashed lines; G-H), depicted for low and high values of β and µ interaction strengths. Grazing rate g=0.00047 d^{-1} (left) and rainfall rate r=600 mm y^{-1} (right); the rest of the parameter values are as in Table 1. In the legends, β units are m^2 g^{-1} and µ is dimensionless.

S3. Additivity of interactions

The additivity between water uptake and each of the two facilitative interactions differed because of their different direct or indirect impacts on soil water level The sum of the partial effects of infiltration amelioration and of water uptake matched their net effect on protégé performance (blue lines in Fig. 3J-L). Both the positive effect of infiltration and the negative effect of water uptake were mediated only by direct changes in soil water content when the grazing protection was not considered (UI model). Conversely, grazing protection was not additive with water uptake (red lines in Fig. 3J-L), because of the nonlinear (indirect) dynamics. Although the grazing protection had no direct effect on water level, it diminished the loss rate of the protégé, which in turn induced non-linear indirect effects through changes on both plants' densities. An increase in grazing decreased the protégé density and thus its water uptake; the increased soil water content led to an increase in the density of the water-limited protégé; this in turn reduced light availability and with it the density of the nurse. These changes would then feed back to the soil water content and the rate of grazing protection on the protégé.

S4. Supplementary figures

S4.1 Difference in the biomass of the protégé between the no-nurse model and the int- or sub-models

The differences between the no-nurse model and the sub-models or int-models, Δp, are depicted to clarify their shapes along the stress gradients. To this end, we report only a few of the panels from Figs. 3 and 4 in the main, namely: compare panels: A,B,D,E,G,H in Figs. 3 and S4; C-J in Figs. 4 and S5.

Notice that Table S4 shows that the differences in the protégé biomass between the no-nurse model and the sub-models were smooth monotonical functions of the stress gradients (Fig. S4A-G), except for the G' model along the grazing stress (Fig. S4H).

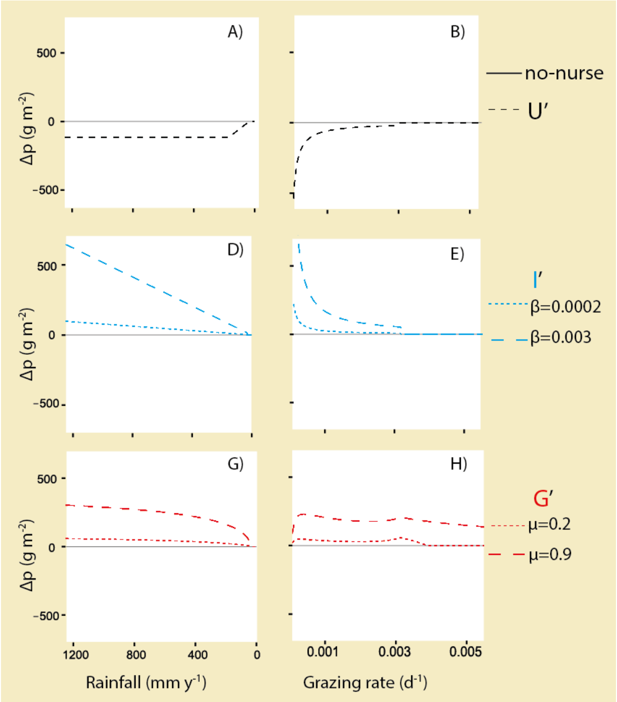

Figure S4. Differences in the biomass of the protégé, Δp, as displayed in Fig. 3, in the 1-int sub-models as a function of water (left) and grazing (right) stress. A-B) U' (black dashed lines), D-E) I' (blued dotted and dashed lines for high and low interaction intensity) and G-H) G' (red dotted and dashed lines for high and low μ interaction intensity). Grazing rate g=0.00047 y^{-1} (left), rainfall rate r=600 mm y^{-1} (right) and nurse density n'=500 g m^{-2} (all panels); the rest of the parameter values are as in Table 1. Notice that the differences in the biomass of the protégé as a function of the nurse biomass are shown in Fig. 3 (right column) and not displayed here (missing panels D,G,I).

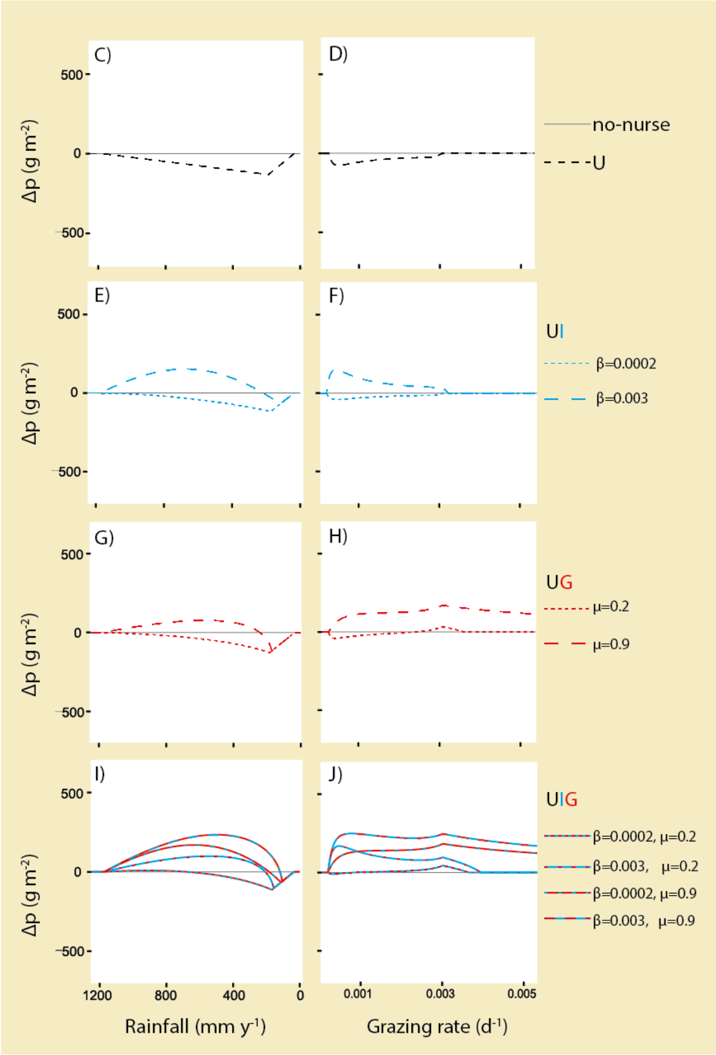

Figure S5. Differences in the biomass of the protégé, Δp, as displayed in Fig. 4, along the water stress (left) and grazing (right) stress gradients for: 1-int U model (black dashed lines; C,D); 2-int models UI (blue dotted and dashed lines; E,F), UG (red dotted and dashed lines; G,H) depicted for low and high values of β and μ interaction strengths; e-int UIG model (intermittent blue-red lines; I,J for the four combinations of low and high values β and μ interaction strengths. Values for grazing rate and rainfall rate are g=0.00047 d^{-1} (left) and r=600 mm y^{-1} (right); the rest of the parameter values are as in Table 1. Notice that the differences in the biomass of the nurse are shown in Fig. 4 (top) and not displayed here (missing panels A,B). In the legends, β units are m2 g-1 and μ is dimensionless.

S4.2 Nurse biomass in all the int-models

For the sake of simplicity, Fig. 4A,B in the main only displayed the change in biomass of the nurse species along the water and grazing stress gradients in the U- model. Panels in Fig. S6 compares this model with that include positive interactions, i.e. UI, UG and UIG. Fig. S6 shows how positive interactions decreased the biomass of the nurse with respect to the U model. Facilitation on the protégé reduced light availability for nurse growth, particularly at low water stress in the UI model (Fig. S6A) and at high grazing stress in UG model (Fig. S6B).

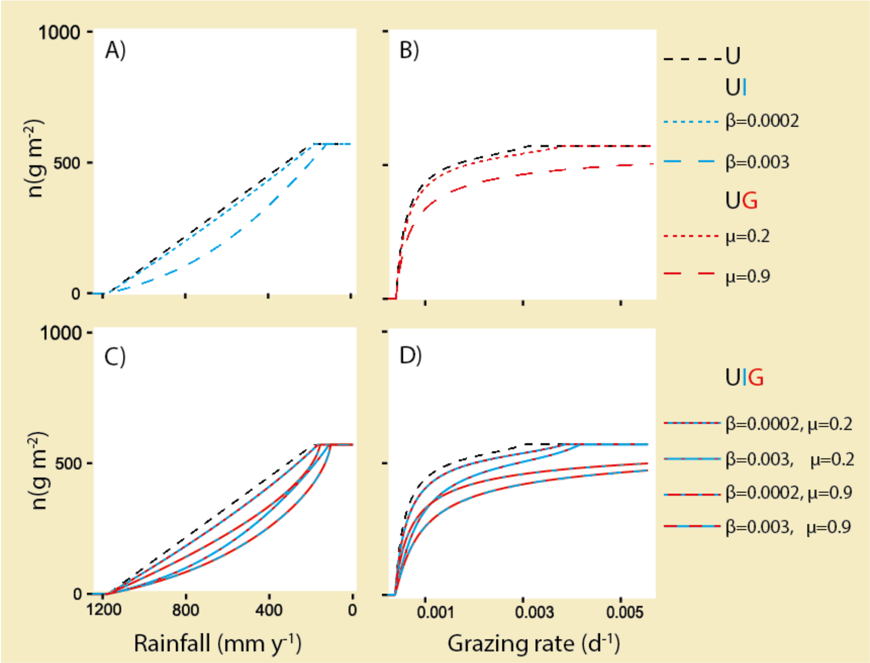

Figure S6. Biomass of the nurse along the water (left) and grazing (right) stress gradients of models U (black dashed lines; A-D), UI (blue lines; A), UG (red lines; B) and UIG (intermittent blue-red lines; C,D). The 2-int models UI and UG are depicted for low and high values of the two interaction strengths (β and μ); 3-int UIG model results are depicted for four combinations of low and high values of β and μ interaction strengths. Values for grazing rate and rainfall rate are g=0.00047 y^{-1} (left) and r=600 mm y^{-1} (right); the rest of the parameter values are as in Table 1. In the legends, β units are m^2 g^{-1} and μ is dimensionless.

S5. Sensitivity analysis.

As a sensitivity test for the representative values of the parameters (Table 1), we modified four key features of the model (three partial interactions strengths and the ratio between the biomasses of the protégé and the nurse) and represented the values of the neighbor indices along the stress gradients (as in Fig. 2). We varied one parameter at a time for the following values (determined for the nurse and the protégé species to coexist in portions of the analysed stress gradients, Fig. S2):

- Ratio of the maximum biomasses of the protégé and the nurse species. Parameter: growth rate of the nurse (γ_n=0.0005, 0.001, 0.003, 0.04 d^{-1}) (Fig. S7).
- Partial interaction strengths:

    - Competition. Parameter: water-to-biomass conversion rate of the nurse (h_n= 0.5, 2, 3.33, 5, 7, 10 m^2 mm g^{-1}) (Fig. S8)

    - Positive interaction mediated by a resource. Parameter: infiltration improvement (β=0.0002, 0.0006, 0.001, 0.002, 0.003, 0.004, 0.005, 0.01 mm d^{-1}) (Fig. S9A,C,D,E,G,H);

    - Positive interaction not mediated by a resource. Parameter: maximum grazing protection (μ=0.01, 0.1, 0.3, 0.5, 0.7, 0.9, 0.99) (Fig. S9B,C,D,F,G,H);

Figs. S7-S9 do not show qualitative changes in the SGH shapes. The only exceptions were UG and UIG at low grazing stress and for parameter values that yield low biomasses of the nurse: low h_n (Fig. S7G,F,D,H, dotted lines) or low γ_n (Fig. S9G,F,D,H, dotted lines). In such cases, the nurse biomass was too low to protect the much larger biomass of the protégé, so the net effect was dominated by water competition. Neighbour effect indices had large negative values for intensity (minimum value -1 in Figs. 7B-D and 9B-D) and importance (Fig. 9F-H).

These results agree with the analysis derived in Appendices S1 and S2

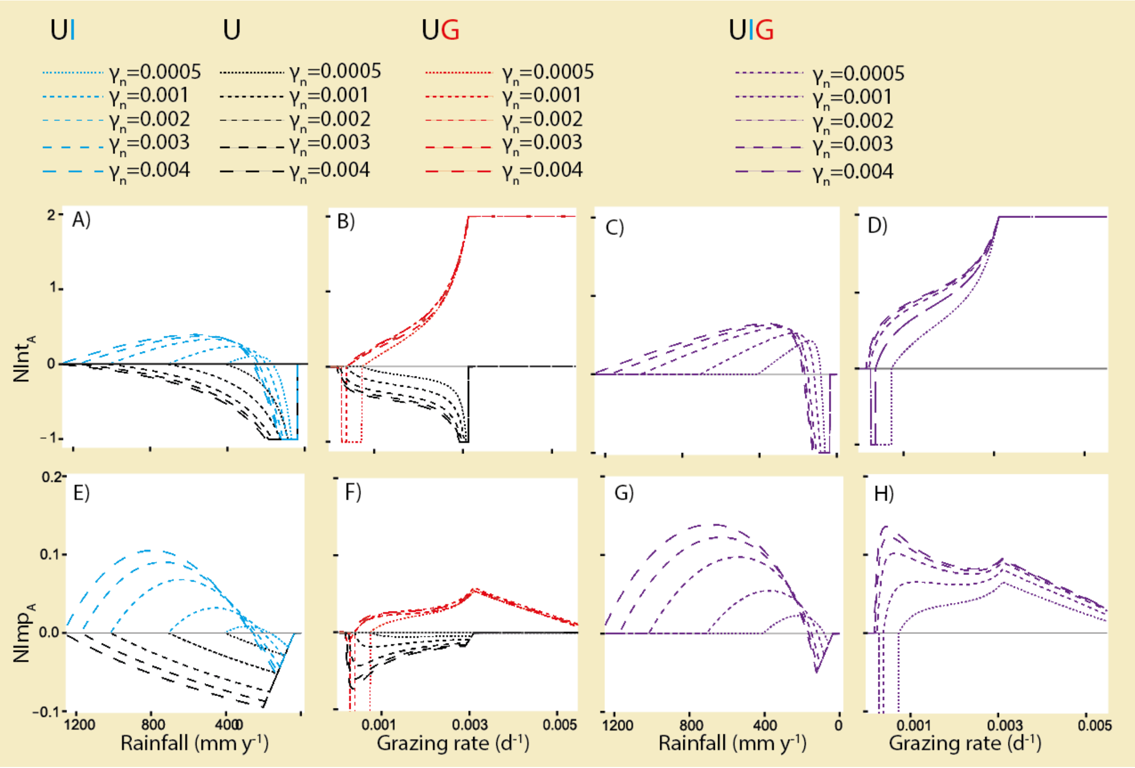

Figure S7. Sensitivity analysis related to Fig. 2 for different values of the growth rate of the nurse (γ_n). Intensity (as given by $NInt_A$, top) and importance ($NImp_A$, bottom) shapes along the relevant stress gradients, for models UI (blue dotted and dashed lines; A,E), UG (red dotted and dashed lines, B,F) and UIG (violet lines, C,D,G,H). The 1-int U model is a particular case of UI and UG models when the facilitative interactions are null (β=0 and g=0). The 2 and 3-int models UI, UG and UIG are depicted for increasing values of the growth rate of the nurse, γ_n, corresponding to increasing intermittency of the blue/red/violet lines. Values for rainfall rate and grazing rate are r=600 mm y^{-1} (B,D,F,H) and g=0.0047 d^{-1} (A,C,E,G); β=0.003 m^2 g^{-1} (A,C,D,E,G,H) and μ =0.5 (B,C,D,F,G,H); the rest of the parameter values are as in Table 1. Notice that the y-axis spans the whole range of the intensity index $NInt_A$, (-1,2) while the importance $NImp_A$ spans only a limited range of values (from -0.1 to 0.2). In the legends, γ_n units are d^{-1}.

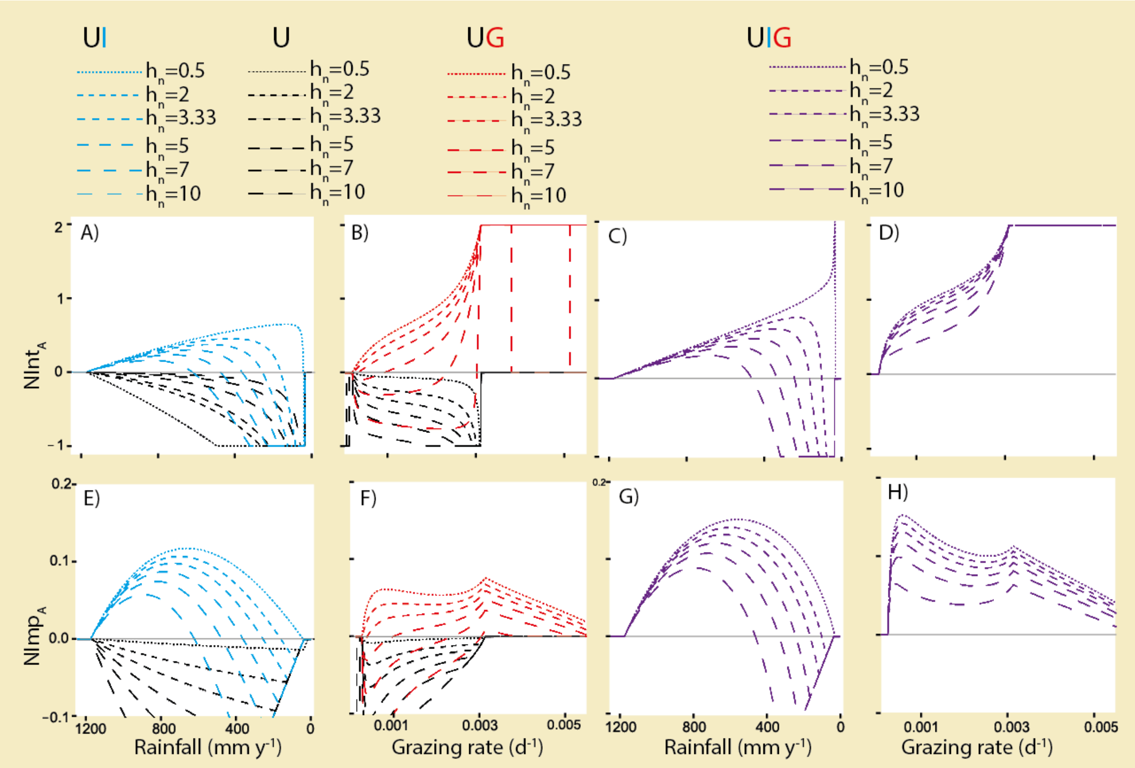

Figure S8. Sensitivity analysis related to Fig. 2, for different water-to-biomass conversion rate of the nurse (h_n). Intensity (as given by $NInt_A$, top) and importance ($NImp_A$, bottom) shapes along the relevant stress gradients, for models UI (blue dotted and dashed lines; A,E), UG (red dotted and dashed lines, B,F) and UIG (violet lines, C,D,G,H). The 1-int U model is a particular case of UI and UG models when the facilitative interactions are null (β=0 and g=0). The 2 and 3-int models UI, UG and UIG are depicted for increasing values of the water-to-biomass conversion rate of the nurse, h_n, corresponding to increasing intermittency of the blue/red/violet lines. Values for rainfall rate and grazing rate are r=600 mm y^{-1} (B,D,F,H) and g=0.00047 d^{-1} (A,C,E,G); β=0.003 mm d^{-1} (A,C,D,E,G,H) and μ =0.5 (B,C,D,F,G,H); the rest of the parameter values are as in Table 1. Notice that the y-axis spans the whole range of the intensity index $NInt_A$, (-1,2) while the importance $NImp_A$ spans only a limited range of values (from -0.1 to 0.2). In the legends, h_n units are m^2 mm g^{-1}

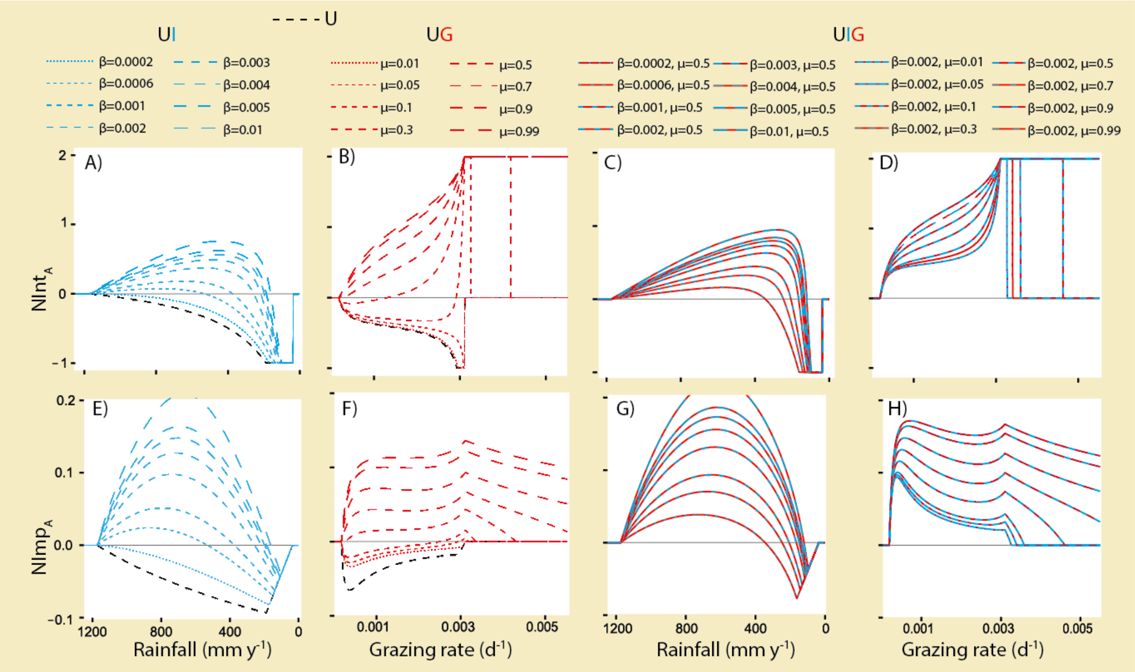

Figure S9. Sensitivity analysis related to Fig. 2 for different values of the positive interaction parameters β and μ. Intensity (as given by NIntA, top) and importance (NImpA, bottom) shapes along the relevant stress gradients, for models UI (blue dotted and dashed lines; A,E), UG (red dotted and dashed lines, B,F) and UIG (intermittent blue-red lines, C,D,G,H). The 1-int U model is a particular case of UI and UG models when the facilitative interactions are null (β=0 and g=0). The 2-int models UI and UG are depicted for a range of values of the two interaction strengths (β and μ); 3-int UIG model results are depicted for combinations of low and high values of β and μ interaction strengths. Values for rainfall rate and grazing rate are r=600 mm y^{-1} (B,D,F,H) and g=0.000047 d^{-1} (A,C,E,G); the rest of the parameter values are as in Table 1. Notice that the y-axis spans the whole range of the intensity index NIntA, while the importance NImpA spans only a limited range of values (from -0.1 to 0.2). In the legends, β units are m^2 g^{-1} and μ is dimensionless.
